# Functional impact of high-altitude hypoxia on central nervous system drug transport: integrated analysis of blood-brain barrier permeability, drug transporter/enzyme expression, and pharmacokinetics

**DOI:** 10.1186/s12987-026-00829-y

**Published:** 2026-06-11

**Authors:** Guiqin Liu, Yue Lin, Lu Tian, Yabin Duan, Qiangqiang Jia, Delong Duo, Qian Wang, Xuejun Wang, Ning Qu, Junbo Zhu, Xiangyang Li

**Affiliations:** 1 School of Pharmacy, Qinghai University, Xining, 810016 China; 2 Affiliated Hospital of Qinghai University, Xining, 810001 China; 3 Qinghai Provincial People’s Hospital, Xining, 810007 China; 4 Red Cross Hospital of Qinghai, Xining, 810000 China; 5 Qinghai Hospital of Traditional Chinese Medicine, Xining, 810000 China; 6 State Key Laboratory of Plateau Ecology and Agriculture, Qinghai University, Xining, 810016 China

**Keywords:** High-altitude hypoxia, Blood-brain barrier, Drug transporters, Drug-metabolizing enzymes, Drug metabolism

## Abstract

**Background:**

The blood-brain barrier is a major obstacle drug transport into the central nervous system. High-altitude hypoxia induces structural and functional alterations in the central nervous system, which in turn can influence drug metabolism and transport throughout the body.

**Methods:**

Rats and human brain microvascular endothelial cells (hCMEC/D_3_) were used as experimental models in this study. The effects of hypoxia on blood-brain barrier structure and function were assessed using Evans Blue staining and confocal laser scanning microscopy. Changes in the expression and activity of drug transporters and drug-metabolizing enzymes under hypoxic conditions were investigated using data-independent acquisition (DIA) proteomic sequencing, RT-qPCR, and western blot. Additionally, pharmacokinetic studies were conducted to evaluate drug concentrations across the blood-brain barrier.

**Results:**

High-altitude hypoxic environments significantly altered the cerebral distribution and trans-blood-brain barrier transport of drug substrates by upregulating the expression of the efflux transporter, ATP-binding cassette subfamily B member 1 (Abcb1), downregulating the expression of the drug-metabolizing enzyme CYP2B1, and increasing blood-brain barrier permeability. Moreover, the prolonged half-life (t₁/₂) and reduced total clearance (CL) observed for drug substrates indicate a significant deceleration in their in vivo metabolism under high-altitude hypoxia.

**Conclusion:**

Our findings preliminarily reveal the differential characteristics of drug metabolism under high-altitude hypoxic environments, implying potential differences in drug disposition between high-altitude and plain human populations. Accordingly, these findings provide novel theoretical insights into the molecular regulatory mechanism of drug metabolism in hypoxic plateau environments, and lay a valuable foundational reference for subsequent basic research on rational drug application in plateau areas.

**Graphical Abstract:**

**Figure:**
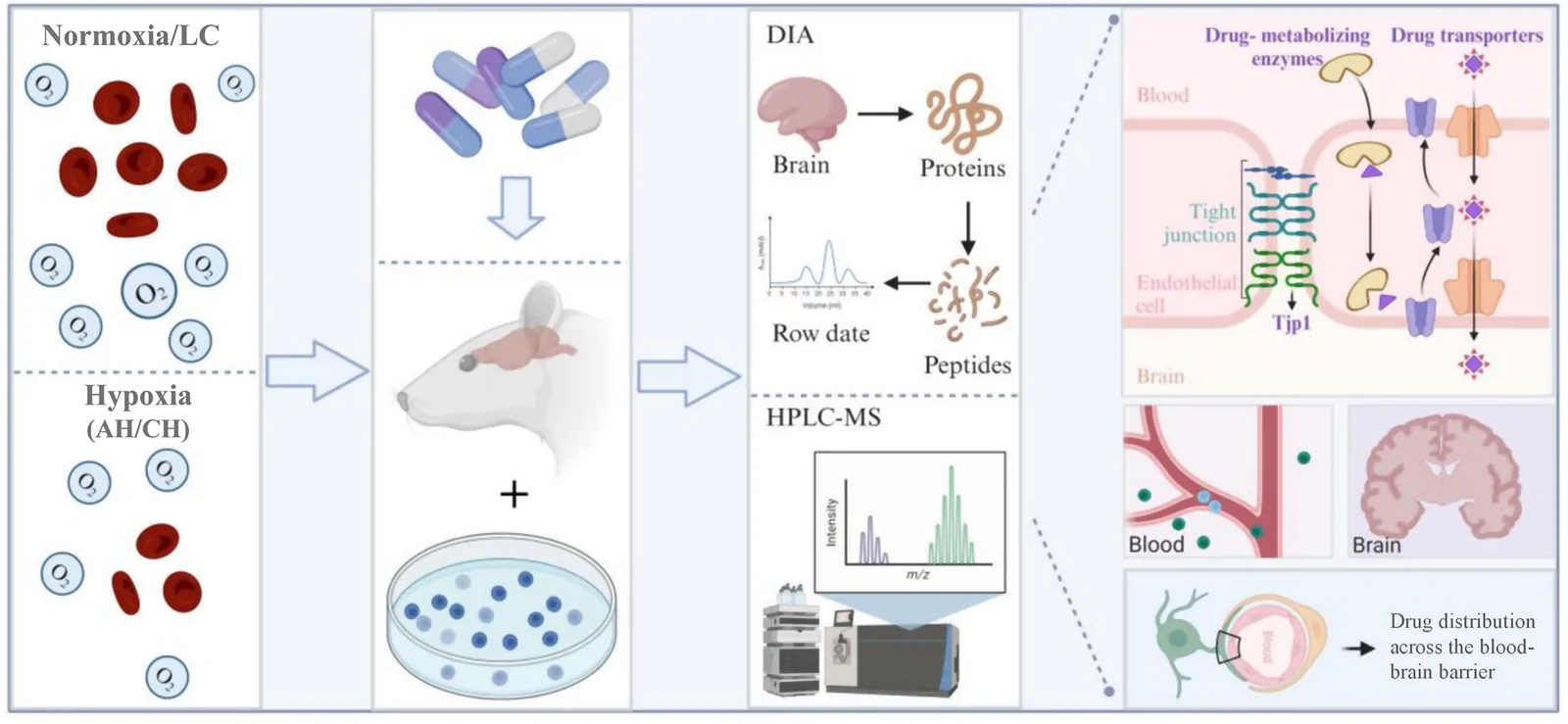

**Supplementary Information:**

The online version contains supplementary material available at 10.1186/s12987-026-00829-y.

## Introduction

Residence at high altitudes may have certain effects on the central nervous system, a highly sensitive system that requires significant amounts of oxygen and energy [1]. The blood-brain barrier plays a vital role in medication uptake and transport, and is indispensable for the treatment of disorders of the central nervous system and the preservation of brain homeostasis. The blood-brain barrier and low intracerebral concentrations prevent some drugs from fully exerting their effects, and this represents a bottleneck in the pharmacological treatment of disorders of the central nervous system, such as cerebral oedema, stroke, Alzheimer’s disease, and epilepsy [2–4]. Cerebral microvascular endothelial cell proliferation is facilitated by changes in the distribution of water and ions in brain tissues, inflammatory factors expression, peripheral immune cell infiltration, and other reactions in the high-altitude hypoxic environment. These changes affect the permeability of the blood-brain barrier, leading to structural and functional dysfunction of the blood-brain barrier. Additionally, damage to the barrier affects the treatment of central nervous system disease, as well as the onset, development, and regression of these diseases; consequently, this affects the drug transporter across the barrier, as well as, drug metabolism [5].

Drugs used to treat diseases of the central nervous system must cross the blood-brain barrier, and reach brain tissues to be effective. Appropriate intracerebral drug concentrations ensure the safety and efficacy of drug therapy, whereas, alterations in in vivo drug metabolism affect drug safety and clinical effectiveness. High-altitude hypoxia-induced changes in the central nervous system affect in vivo drug metabolism. Drugs such as phenobarbital, phenytoin sodium, pethidine, diazepam, and buspirone have been shown to exhibit slowed in vivo metabolism under high-altitude hypoxia [6, 7]. It is recommended that, drug use in plateau areas be different from that in plain areas, and that drug dosage and administration time be adjusted to a certain extent, however, the brain-blood drug partition ratio (*C*_brain_/*C*_plasma_) and the blood-brain barrier permeability coefficient (Kp_(AUCbrain/AUCplasma)_), have received less study attention. Clinical studies have assessed drug central nervous system pharmacokinetics mainly using *C*_brain_/*C*_plasma_ and Kp_(AUCbrain/AUCplasma)_. Drug central nervous system pharmacokinetics under high-altitude hypoxic conditions need to be further investigated, this will be essential for assessing drug transport across the blood-brain barrier, as well as for assessing drug metabolism.

There may be some limitations to the use of the *C*_brain_/*C*_plasma_ and Kp_(AUCbrain/AUCplasma)_ as indicators for the logical assessment of central nervous system-related disease treatment in plateau areas. Aside from the inherent characteristics of the medications, other significant elements influencing the therapeutic effects of drugs, include the degree of barrier integrity, and the activities of drug transporters and drug-metabolizing enzymes within the barrier. Drug transporters expressed in the blood-brain barrier include those of the ATP-Binding cassette transporter superfamily (ABC families) and solute carrier family transporters (SLC families), with Abcb1, ATP binding cassette subfamily G member 2 (Abcg2), and solute carrier organic anion transporter family member 1A2 (Slco1a2), exhibiting the highest expression. Additionally, drug-metabolizing enzymes include members of the CYP1, CYP2, and CYP3 families, with CYP2B6, CYP2D6, and CYP3A4, exhibiting the highest expression. These transporters and metabolic enzymes co-regulate endogenous substance/drug metabolism and transport, which influence drug effectiveness [8–11]. However, the interspecies correspondence between these key regulatory molecules in rats and humans is not fully understood. For instance, rat P-gp (Abcb1) is co-encoded by two genes, Abcb1a and Abcb1b, whereas human P-gp is solely encoded by an ABCB1 gene. Rat Abcg2 shares high homology with human ABCG2, with relatively conserved biological functions. In addition, rat CYP3A1, CYP2B1 and CYP2D1 are homologous metabolic enzymes to human CYP3A4, CYP2B6 and CYP2D6, respectively. However, these isoforms exhibit prominent interspecies differences in substrate selectivity, catalytic activity and regulatory mechanisms of expression. Given that a rat model was adopted in this study, the aforementioned interspecies discrepancies were fully considered in the subsequent analysis and in the discussion of result. The potential influences of such differences on the expression profiles of drug transporters and metabolic enzymes in the blood–brain barrier was clarified to ensure the rigor and rationality of the research conclusions. Our previous study revealed that protein and mRNA expression of Abcg2 was elevated and expression of Abcb1, CYP2B1, and CYP3A1 was reduced in rat liver under plateau hypoxia [12, 13], although little research has investigated how drug transporters and metabolizing enzymes are expressed in the blood-brain barrier under high-altitude hypoxia.

**Fig. 1:**
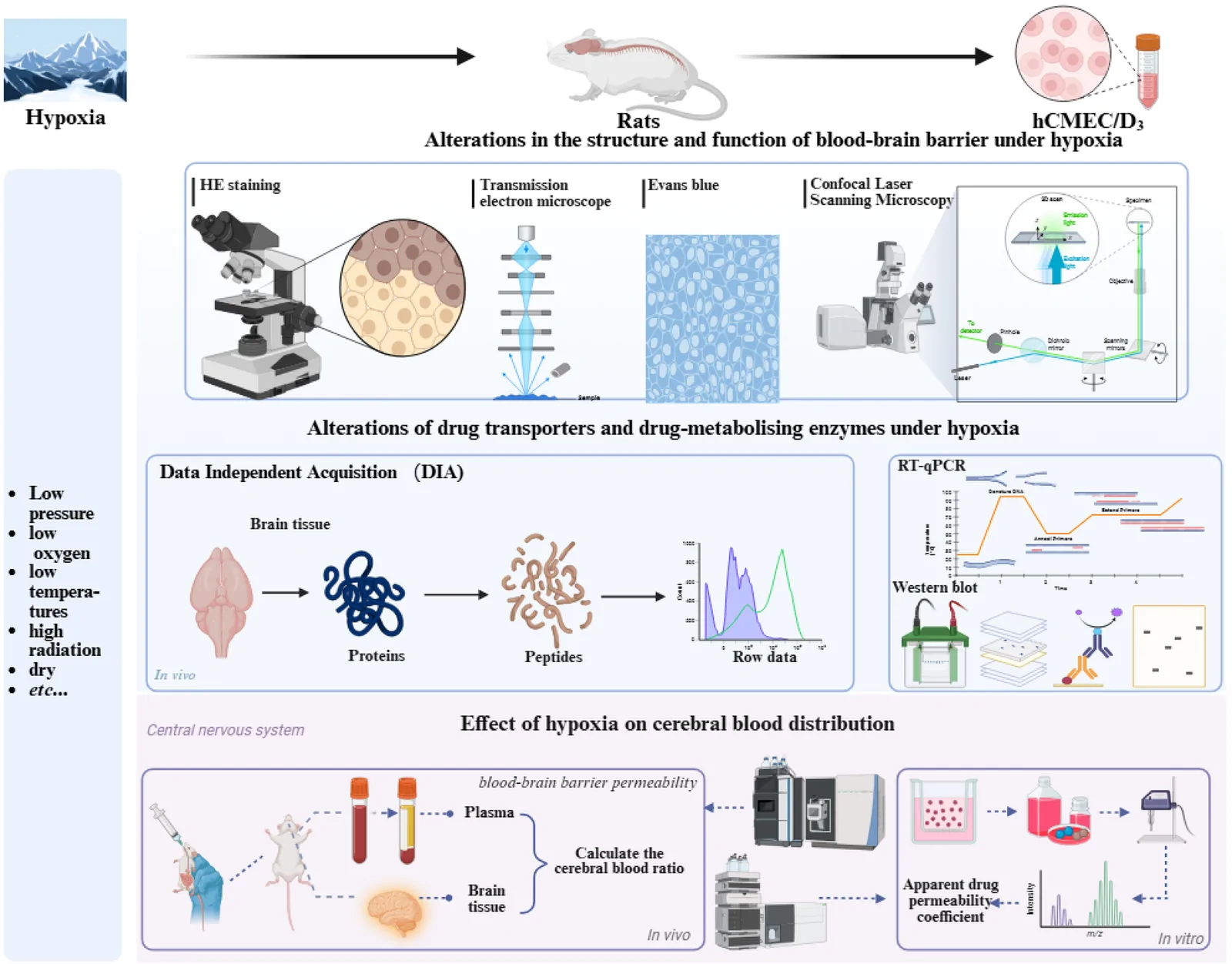
Research framework of this study

This study combines plateau hypoxia, blood-brain barrier, and drug metabolism with rats and hCMEC/D_3_ expressed at the blood-brain barrier, reveals the changes of major drug transporters and metabolism enzymes in the blood-brain barrier in the plateau environment, study of pharmacokinetic characteristics of rat plasma and brain tissue, and preliminary investigation of intrinsic mechanism of high-altitude hypoxic environment on drug transport across blood-brain barrier. Exploring the effects of changes in the blood-brain barrier, drug transporters, drug-metabolizing enzymes, and drugs across the blood-brain barrier on drug metabolism under high altitude hypoxia, to provide a new theory for studying drug metabolism in the high-altitude hypoxia, and to provide a reference for the clinical rational use of drugs used to treat central nervous system disorders at high-altitude hypoxia. The experimental framework of this study is shown in Fig. 1.

## Materials and methods

### Animals and cell culture

Adult Sprague-Dawley rats (male, 180–220 g) were purchased from the Laboratory Animal Center of the Department of Medicine at Xi’an Jiaotong University, License No. SCXK (Shaanxi) 2019–0008. The facility kept a 12 h light/dark cycle, a constant temperature of 24 °C, and standard living circumstances for the animals, with the rats having unlimited access to food and water. Prior to modeling, all rats were randomly allocated into three experimental groups using a stratified randomization procedure based on body weight to ensure baseline homogeneity. All experimental procedures were performed following the guidelines of the Ethics Committee for Animal Experiments, and the protocol was approved by the Animal Ethics Committee of Qinghai University (Permit NO. 2022–24). Rat was modeled in plains and plateau field environments, samples were collected at the Medical College of Xi’an Jiaotong University (Xi’an City, Shaanxi Province, altitude: 390 m, PaO_2_: 20.2 kPa) for the low-altitude control group (LC), and the Huashixia Town Health Hospital (Maduo County, Guoluo Tibetan Autonomous Prefecture, Qinghai Province, China, altitude: 4,300 m, PaO_2_: 12.4 kPa) for the high-altitude acute hypoxia group (AH, low-altitude rats were transported to Maduo County to live 3 days) and high-altitude chronic hypoxia group (CH, low-altitude rats were transported to Maduo County to live 30 days). All experimental conditions were identical, except for the location for model was establishment.

hCMEC/D_3_ were purchased from the Yaji Biological (Shanghai, China). After optimum cell culture, the control group was maintained under normoxic conditions, while the 2, 5, and 10% hypoxia groups were exposed to the respective oxygen concentrations for 24 h in a tri-gas incubator (YCP-30SQ, China) set at 37 °C with a relative humidity of 70%-80%. Additionally, cells in the 2% hypoxia group were subjected to hypoxia for varying durations (2, 6, 12, 24, and 48 h). The cells were cultured in medium containing 10% fetal bovine serum and ECM, and incubated at 37 °C in a 5% CO₂ atmosphere with 90% humidity.

### Brain histopathological morphometry

The rats were anesthetized via the intraperitoneal injection of 20% urethane (1 g/kg). The abdominal cavity of each animal was exposed with surgical scissors and forceps to reveal the heart. A needle was inserted into the right atrium, and the sinusoidal vein was clamped with arterial forceps. The animals were then perfused with saline until their livers turned white and then with 4% paraformaldehyde until their livers hardened. After complete fixation, the head of the rat was cut off, and the brain was removed. The rat brains were soaked in 4% paraformaldehyde for 1 day, then dehydrated and paraffin-embedded. The pathological changes of the brain tissues were then examined under a light microscope (BA210 Digital, China).

### Transmission electron microscope

To assess the effects of hypoxic plateau conditions on the blood-brain barrier, rat brain tissues were processed for transmission electron microscopic analysis to determine the impact of plateau hypoxic conditions on the blood-brain barrier. The samples were pre-fixed with glutaraldehyde, dehydrated with acetone, infiltrated, embedded with a dehydrating agent and Epon812 embedding agent, sliced into 60-nm sections using an ultrathin sectioning machine (UC7rt, Germany), and analyzed using a JEM-1400FLASH transmission electron microscope (JEOL, Japan).

### Fluorescein sodium and Evans blue staining

The Evans blue staining (2%, E6135, Macklin, Shanghai, China) solution was administered to the rats tail vein injection prior to dissection. Following a two-hour anesthesia period, the rats were secured onto the operation platform, their chest cavities were opened, and physiological saline was infused into their hearts. Then, brain tissue were removed, and the left hemisphere of each brain was fixed in paraformaldehyde. Thereafter, the fixed brain tissues were transferred to a refrigerator at −80 °C. Upon removal, the brain tissue was trimmed and cut flat, submerged in a 15% sucrose solution, and dehydrated in a refrigerator at 4 °C. The frozen and embedded dehydrated tissue was then taken and sliced after it turned white and solidified. Sections were imaged using an AI confocal laser scanning microscope (Nikon, Japan). Image processing and quantification of the average fluorescence intensity were performed using the Image-J software (National Institutes of Health, USA). The right hemispheres was kept in liquid nitrogen for measurement, the amount of Evans blue in the brain tissue was determined by weighing the tissue, adding formamide, centrifuging it for 15 min at 3,000 × *g*, for supernatant extraction, then, absorbance at 630 nm was measured using an enzyme marker.

### Cell viability assay

The viability of hCMEC/D_3_ under different hypoxic conditions - varying oxygen concentrations and exposure durations (2, 5, and 10% O₂ for 24 h, and 2% O₂ for 2, 6, 12, 24, 48 h)—was evaluated using a CCK-8 assay kit. After cell treatment, the cell plate’s supernatant was removed and incubated at 37 °C with CCK-8 working solution, finally, optical density (OD) at 450 nm was measured using an enzyme labeller (Thermo, USA).

### Sodium fluorescein and trans-epithelial electrical resistance assays

After the cultivation process, hCMEC/D_3_ were inoculated into transwell chambers, exposed to varying hypoxic conditions and durations until fusion, and then treated with 0.1 mg/mL of fluorescein isothiocyanate-dextran (60842–46-8, Merck, Darmstadt, Germany) was added. After 15 min, the medium was removed from the transwell inserter, and fluorescence signals (Ex = 485 nm, Em = 538 nm) were observed to analyze the permeability of the hCMEC/D_3_. The trans-epithelium electrical resistance was measured using the other group of cells. After soaking the electrodes of the RE1600 meter (Kingtech, China) in HBSS for 20 min, the medium in the culture plate was removed, and pre-warmed HBSS was added, 0.5 mL per well on the apical side of the filtration membrane (AP) side, and 1.5 mL per well on the basolateral side of the cell (BL) side, and the cells were equilibrated at 37 °C for 20 min. Following removal of any contaminants from the cell surface, HBSS was removed and replaced with pre-heated HBSS. The integrity and permeability of the cell layer were assessed by measuring the transmembrane resistance.

### DIA and bioinformatics analysis

Brain tissues collected from rats in three groups were subjected to total protein extraction. Proteomic sequencing procedures included protein extraction and digestion, high pH reversed phase separation, data-dependent acquisition (DDA) qualitative library construction, library searching, and DIA data collection. These procedures were performed following previously published methods [14]. Spectronaut X (Biognosys AG) was used to analyze raw data and search the database using the rat genome Ensembl_release106. Protein characterization necessitates that the filtered peptides and proteins be utilized for quantitative analysis and that the precursor threshold 1.0% False Discovery Rate (FDR) and protein threshold 1.0% FDR are satisfied at the peptide and protein levels, respectively, in order to guarantee the validity of the results. Ultimately, a protein quantification screen was conducted using the average of the peak regions of the first three MS1 peptides with an FDR smaller than 1.0%.

We primarily examined drug transporters and metabolizing enzymes with FDR less than 1.0%, and we thoroughly examined proteins linked to drug metabolism in rat brain tissues. Student’s T test was performed in R to obtain *p*-values, and proteins with Qvalue < 0.05 and |log2(1.5)| >0.58 were identified as differentially expressed proteins dep, and proteins with significant differences in the groups were screened, for analysis of differential expression, *p*-values were adjusted for multiple testing using the Benjamini-Hochberg procedure, thereby ensuring strict FDR control. An online venn and sankey, bubble, and volcano plot analyses of the association between the proteins in each sample group were conducted using the R package and the omicshart software. Subsequently, dep were annotated using GO pathways, abd the Q values of the pathways were determined using R and the hypergeometric test. GO pathways with Q < 0.05 were considered statistically significant and described the target gene’s molecular function, cellular component, and biological process, respectively. In addition, traditional enrichment analytical approaches do not allow for effective information mining on microeffect genes, thus, to compensate for this and other shortcomings, we performed a Gene Set Enrichment Analysis (GSEA analysis) based on the whole protein set, applied the permutation test to calculate the significance *p*-value, and corrected the normalized Enrichment Score value using multiple tests to obtain the FDR value, which provides a more comprehensive explanation of the regulatory role of the GO terms. Finally, interaction network relationship maps between candidate proteins and proteins were created using Cytoscape and the STRING proteins interaction database. Then, interactions between the proteins and the networks that resulted from these interactions were then investigated.

### Western blotting

The RIPA lysate was used to extract total proteins from brain tissues and hCMEC/D_3_, and a BCA kit was used to determine the protein content. Western Blotting was carried out according to standard procedures using 10% SDS polyacrylamide, and PVDF membrane. The primary antibodies were used in this experiment included those against Abcb1 (1: 1,000, AB170904, Abcam, Cambridge, UK), Abcg2 (1: 1,000, AB207732, Abcam, Cambridge, UK), Slco1a2 (1: 5,000, ABS118501, Absin, Shanghai, China), CYP2B6 (rat homologue of CYP2B1) (1: 2,000, NBP2-92746, Novus Biologicals, Colorado, USA), CYP2D6 (rat homologue of CYP2D1) (1: 2,000, AB230472, Abcam, Cambridge, UK), CYP3A4 (rat homologue of CYP3A1) (1: 1,000, AB3572, Abcam, Cambridge, UK), Tjp1 (1: 5,000, 000033004, Novus Biologicals, Colorado, USA), and β-actin (1: 2,000, YT0099, Immunoway, Texas, USA). The secondary antibody used was goat anti-rabbit IgG (1: 10,000/1: 20,000, AB205718, Abcam, Cambridge, UK). A multifunctional imager (Bio-Rad, Hercules, CA, USA) was used to detect the protein bands, and Image J was used to analyze the data in grayscale.

### Quantitative real-time PCR (RT-qPCR)

Following extraction of total brain tissues and cellular RNA according to the instructions of the kit manufacturer (Q41206; TransGen, Beijing, China), the quality of the RNA solution was evaluated using a NanoDrop 2000c spectrophotometer (Thermo, USA). The transscript one-step gDNA removal and cDNA synthesis supermix kit (Q31123, TransGen, Beijing, China) was used to do synthesize reverse cDNA synthesis. The mRNA expression of Abcb1, Abcg2, Slco1a2, CYP2B6, CYP2D6, CYP3A4, and Tjp1 in the blood-brain barrier and hCMEC/D_3_ was detected by Roche light cycler 96 real-time fluorescence quantitative PCR instrument (Roche, Switzerland). Quantitative analysis of the experimental data was performed using the 2^-ΔΔCt^ method, and supporting information Tables S1 and S2 contain a list of all the primers.

### Pharmacokinetic study

To evaluate the pharmacokinetic alterations in the blood-brain barrier under high-altitude hypoxia, the following groups were included: low-altitude control group, high-altitude acute hypoxia group, and high-altitude chronic hypoxia group. Rats were weighed and administered different substrates via gavage after a 12 h fast and water deprivation period. The substrate drug propranolol hydrochloride of Abcb1 was given by gavage at a dose of 100 mg/kg and decapitation for blood and brain tissues were collected at 0.08, 0.25, 0.5, 1, 2, 4, 6, 8, 12 and 24 h after administration. Verapamil hydrochloride, a drug substrate drug of CYP2B1, was administered via gavage at a dose of 50 mg/kg and decapitation for blood and brain tissues were collected at 0.16, 0.5, 1, 2, 3, 4, 6, 8, 12 and 24 h after administration. Zolmitriptan, a drug substrate of Slco1a2, was administered via gavage 100 mg/kg and decapitation for blood and brain tissues were collected at 0.08, 0.17, 0.5, 1, 2, 3, 4, 6, 8, and 10 h after administration. After that, evaluate in vivo drug metabolism changes in rats based on changes in pharmacokinetic parameters of propranolol hydrochloride, verapamil hydrochloride and zolmitriptan in the groups. All blood samples were centrifuged for 15 min at 3,000 × *g*, and the supernatants were retrieved and preserved at −20 °C, brain tissues were preserved in liquid nitrogen for subsequent analyses.

#### Internal standard solution and sample handling

The mesylate hydrochloride, ubenimex, and sumatriptan succinate control products were carefully weighed, dissolved in methanol, and used to set the volume. Before being accurately measured and diluted with methanol to create an internal standard working solution of 100 ng/mL for sample determination, the internal standard stock solution of 1.00 mg/mL was created.

Methanol was applied to each group of plasma and brain tissue samples in order to normalize the proteins, and then the mixtures of methanol, internal standard (IS), and either plasma or brain tissue were swirled for thirty seconds. After centrifugation at 14,000 × *g* for 12 min at 4 °C, the supernatants were extracted using 0.22 μm organic filter membranes. Thereafter, the filtrates were then placed in injection bottles and the results were examined using ultra performance liquid chromatography-mass spectrometry (UPLC-MS) (Thermo Scientific Ultimate 3,000 and Q-Exactive Focus MS, Sunnyvale, CA, USA).

#### Chromatographic conditions

The analysis was performed using a UPLC-MS, chromatographic column: thermo Hypersil GOLD C18 column (2.1 mm × 50 mm, 1.9 μm) at a column temperature: 35 °C. Propranolol and its internal standard, mexiletine hydrochloride, were isocratically eluted following this method with acetonitrile used as mobile phase A and a 0.1% formic acid aqueous solution used as mobile phase B, with the ratio of A: B = 61%:39%, the flow rate was 0.25 mL/min; the injection volume was 5 μL. The analysis of verapamil and its internal standard, ubenimex, was performed with mobile phase A as 0.1% formic acid + 5 mM ammonium formate aqueous solution and mobile phase B as acetonitrile, with an elution gradient of 0 min, 25% B; 4 min, 90% B; 5 min, 90% B; 6 min, 25% B; and 8 min, 25% B; the flow rate of 0.25 mL/min; and the injection volume was 2 μL. Zolmitriptan and its internal standard sumatriptan succinate were separated in a gradient elution with 0.1% water formic acid + 10 mM ammonium formate aqueous solution used as mobile phase A and methanol used as mobile phase B. The elution gradient was as follows: 0 min, 5% B; 4 min, 90% B; 5 min, 90% B; 6 min, 5% B; 8 min, 5% B; the flow rate was 0.25 mL/min; and the injection volume was 2 μL.

#### Mass spectrometric conditions

Mass spectral data were obtained by analyzing samples in the positive, Full MS mode using an ESI ion source. Mass spectrometric parameters as follows: sheath gas flow rate of 45 arb; auxiliary gas flow rate of 15 arb; capillary temperature of 320 °C; resolution of 70,000 voltage of 3.8 kV (positive mode) or −3.1 kV (negative mode). The extracted ion mass-to-charge ratios [M + H] (m/z) were 260.1645 and 180.1382 for propranolol hydrochloride and mexiletine hydrochloride, respectively. The extracted ion mass-to-charge ratios [M + H] (m/z) were 455.2904 and 309.1809 for verapamil hydrochloride and ubenimex, respectively. The extracted ion mass-to-charge ratios [M + H] (m/z) for zolmitriptan and sumatriptan succinate were 288.1709 and 296.1427, respectively.

#### Method validation

All methods used for biological sample analysis were validated against the 2020 edition of the “Chinese Pharmacopoeia guidelines” and relevant US FDA guidelines for the established UPLC-MS methods. Validation parameters included selectivity, standard curve performance, accuracy, precision, recovery, and stability.

#### Pharmacokinetic assays

Plasma and brain tissue samples were collected from each group of rats, and the concentrations of propranolol hydrochloride, zolmitriptan, and verapamil hydrochloride were determined, respectively. Pharmacokinetic analysis was performed using DAS 2.0 software based on a noncompartmental model fitting of the plasma concentrationtime data. The core pharmacokinetic parameters were obtained and compared among groups, including clearance (CL/F), elimination halflife (*t*₁/₂), peak concentration (Cₘₐₓ), time to peak (Tₘₐₓ), area under the curve (AUC), apparent volume of distribution (V_d_/F), and mean residence time (MRT). The relevant calculation formulas were defined as follows: The trapezoidal method was adopted to calculate ${\rm{AU}}{{\rm{C}}_{{\rm{0 - }}t}}{\rm{ = }}\sum\limits_{i{\rm{ = 1}}}^n {{{{C_{i{\rm{ - 1}}}}{\rm{ + }}{C_i}} \over {\rm{2}}}} \times ({t_i}{\rm{ - }}{t_{i{\rm{ - 1}}}}),$ and AUC_0-∞_ was extrapolated with the terminal elimination rate constant λz: ${\rm{AU}}{{\rm{C}}_{{\rm{0 - }}\infty }}{\rm{ = AU}}{{\rm{C}}_{{\rm{0 - }}t}}{\rm{ + }}{{{C_{{\rm{last}}}}} \over {{\lambda _z}}}$, the ${t_{{\rm{1/2}}}}$ was calculated as ${t_{{\rm{1/2}}}}{\rm{ = }}{{{\rm{0}}{\rm{.693}}} \over {{\lambda _z}}}$, CL/F was derived from ${\rm{CL}}/{\rm{F}} = {{{\rm{Dose}}} \over {{\rm{AU}}{{\rm{C}}_{0 - \infty }}}}$, V_d_/F was obtained by ${\rm{Vd/F = }}{{{\rm{CL/F}}} \over {{\lambda _z}}},$ the area under the first moment curve from zero to the last time point (AUMC₀₋ₜ) was calculated using ${\rm{AUM}}{{\rm{C}}_{{\rm{0 - }}t}}{\rm{ = }}\mathop \sum \limits_{i{\rm{ = 1}}}^n {{{t_{i{\rm{ - 1}}}}{C_{i{\rm{ - 1}}}}{\rm{ + }}{t_i}{C_i}} \over {\rm{2}}}{\rm{ \times (}}{t_i}{\rm{ - }}{t_{i{\rm{ - 1}}}}{\rm{)}}$, and MRT was obtained by ${\rm{MRT = }}{{{\rm{AUM}}{{\rm{C}}_{{\rm{0 - }}\infty }}} \over {{\rm{AU}}{{\rm{C}}_{{\rm{0 - }}\infty }}}}$, Cₘₐₓ and Tₘₐₓ were the maximum measured concentration and its corresponding time, respectively. In addition, the blood-brain barrier permeability was evaluated by calculating the *C*_brain/_*C*_plasma_ and Kp_(AUCbrain/AUCplasma)_. These indices were used to assess drug transport across the blood-brain barrier and to analyze the effects of high‑altitude hypoxia on trans-blood-brain barrier drug transport. Finally, a preliminary mechanistic interpretation was provided based on alterations in the expression and activity of metabolic enzymes and transporters.

### hCMEC/D_3_ cell transport across the blood-brain barrier

#### Single-layer cell model construction

To establish the hCMEC/D_3_ single-cell layer model, the hCMEC/D_3_ were inoculated into transwell chambers, and type I collagen was added to the apical and basolateral sides of the medium, and the cells were grown using EGM-2 endothelial cell-specific medium. An electron microscope was used to scan the cell monolayer and to carry out cell morphological observations. A resistivity meter was used to measure the transmembrane resistance of the monolayer membrane for the assessment of the integrity and density of the monolayer model and for determining whether the state of the cells was appropriate for subsequent investigations.

#### hCMEC/D_3_ cell uptake and transport assays

Uptake and translocation experiments were performed using previously described methods [15]. Normoxia was used as the control group, and hypoxia groups for Abcb1 and Slco1a2 were established, resulting in three groups in total. Cells in the hypoxia group were treated based on the optimal oxygen concentration and treatment duration (2% O_2_, 2 h) identified through the previously conducted tests. After hypoxia treatment of hCMEC/D_3_, 2 mL of HBSS solution containing Abcb1, Slco1a2 substrate drugs propranolol hydrochloride and zolmitriptan were added, respectively, and after a certain amount of incubation time, the experiment terminated, the cells were ultrasonically pulverized, and the content was determined by HPLC (Thermo Scientific Ultimate 3,000 Sunnyvale, CA, USA) to examine the drug uptake at 30, 60, 90 and 120 min. After subjecting hCMEC/D_3_ cells to the hypoxia treatment, substrate drug transport experiments were carried out from the basolateral (BL) side of the cell to the apical plane (AP) side, as well as from the AP side to the BL side, respectively. 100 μL of samples were sampled at 30, 60, 90, and 120 min, and their drug concentrations were determined via HPLC. To evaluate drug transport, the apparent drug permeability coefficient through the cell monolayer was determined using the formula, Papp = (dQ/dt/C_0_)/(A×C_0_).

Where dC/dt (μmol/s) is the rate at which the compound appears in the receiver chamber, A (cm^2^) is the surface area of the transwell polycarbonate membrane (1.12 cm^2^), and C_0_ (μmol/L) is the initial concentration of drugs.

#### Cell sample processing

The HBSS solution samples were treated with acetonitrile treatment to denature the proteins. Then, they were sonicated for 30 min, centrifuged for 12 min at 3,000 × *g*, and the filtrate was collected and poured into injection vials for HPLC analysis.

#### Cell chromatographic conditions

The chromatographic separation was carried out using a Thermo Hypersil GOLD C18 250 mm × 4.6 mm, 0.5 μm column (Thermo Fisher, Sunnyvale, CA) at a column temperature of 35 °C, a flow rate of 1 mL/min, an injection volume of 10 μL,and an analysis time of 120 min. The mobile phase for propranolol hydrochloride elution was acetonitrile + a 0.05 mol/L potassium dihydrogen phosphate solution (60–40); while that for zolmitriptan elution was acetonitrile + a 0.05 mol/L potassium dihydrogen phosphate solution (triethylamine adjusted pH = 5.5 ± 0.5) (20–80).

### Statistical analysis

All data were analyzed using SPSS 27.0 software, and quantitative results were presented as mean ± standard deviation (SD). An Independent sample t-test was applied for comparison between two groups. One-way analysis of variance (ANOVA) was used for multi-group comparisons, and repeated-measures ANOVA was used for data with repeated measurements. Post hoc multiple comparisons were conducted based on the LSD correction according to the experimental design. All statistical tests were two-tailed, and a value of (*p* < 0.05) was considered statistically significant.

## Results

### Alterations in rat brain tissue structure under high-altitude hypoxia

**Fig. 2:**
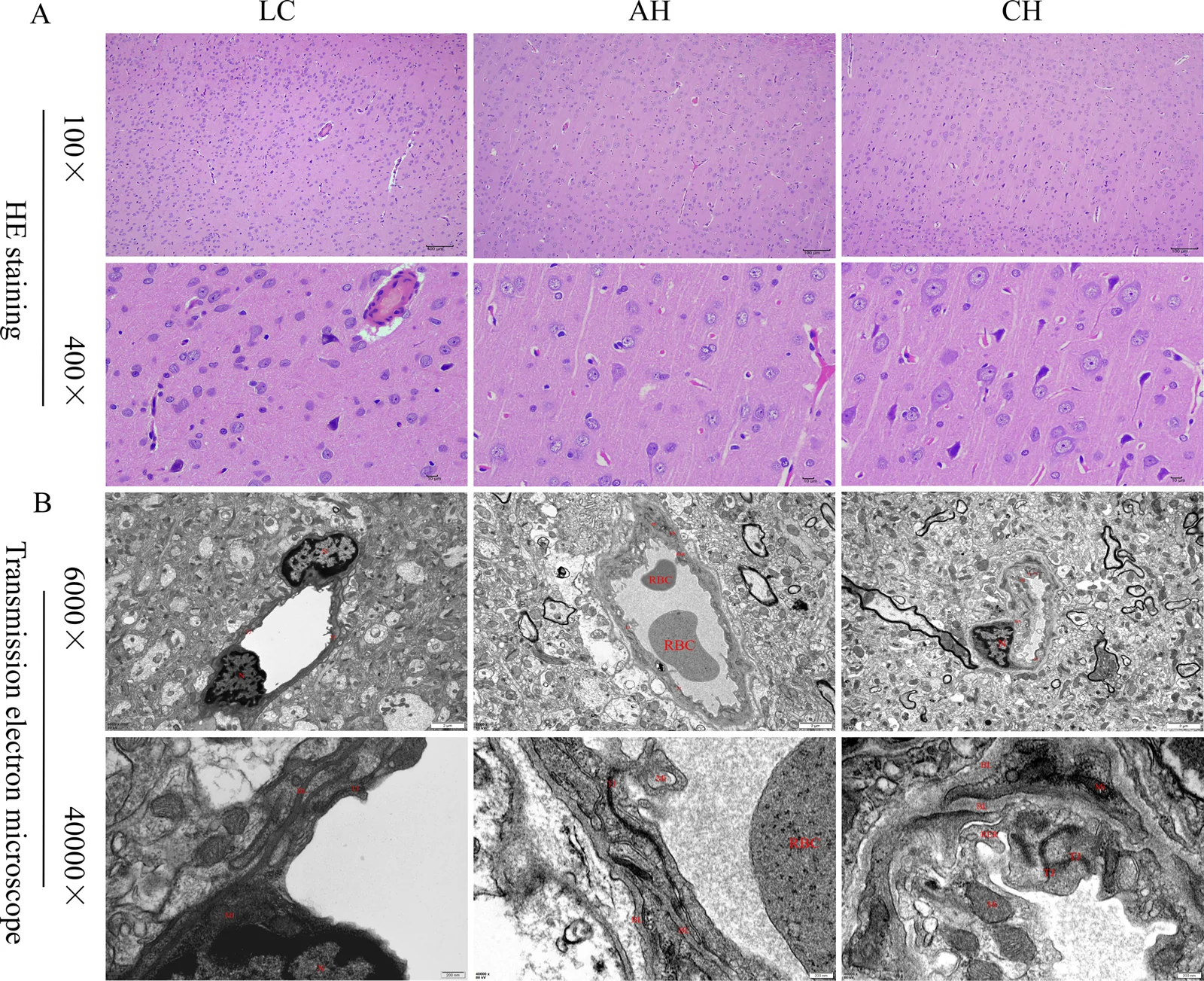
Changes in the structure and function of rat brain tissues under high-altitude hypoxia. Note: **A**: Results of HE staining. **B**: Results of transmission electron microscope. LC, AH, CH refer to the low-altitude control group, high-altitude acute hypoxia group, and high-altitude chronic hypoxia group, respectively

Exposure to high-altitude hypoxia induced injury in rat brain tissues, with the severity of the damage increasing with altitude. Our HE analysis revealed that the brain tissues of the low-altitude control group had a soft meningeal structure that was intact, with the normal vascular distribution and no visible inflammatory exudation, and the cortical area’s cell layers were more neatly arranged, and the morphology of the neuronal was normal. The high-altitude acute hypoxia group had sporadic appearances of dark-colored neurons, a reduction in cell size and color, blurring of the internal cell structure, and the presence of axon-like structures in the cytosolic tail, and there was no overt gliocyte hyperplasia or inflammatory cell infiltration in the high-altitude chronic hypoxia group, but there were sporadic observations of dark-colored neurons (Fig. 2A). Electron microscopic data revealed changes in the tight junction architectures of blood-brain barrier. Endothelial cells in rats in the low-altitude control group exhibited typical tight junctions (TJ), basement membranes (BL), nuclei (N), mitochondria (Mi), and several structural features. The high-altitude acute hypoxia group’s vascular endothelial cells were shaped like a pike, facing the lumen and attached beneath the basement membrane, they had continuous basement membranes and intact nuclear membranes, but there were also noticeable oedematous perivascular phenomena, swollen intracytoplasmic mitochondria, and noticeably open tight-junctions. In the high-altitude chronic hypoxia group, vascular endothelial cells were seen to be pike-shaped, with normal cytoplasmic mitochondria, but with slightly open tight-junctions, increased pericytes, reduced dense material content minor dilatation of the rough endoplasmic reticulum, and enhanced autophagy (Fig. 2B).

### Variations in the permeability of the blood-brain barrier and hCMEC/D_3_ under hypoxia

**Fig. 3:**
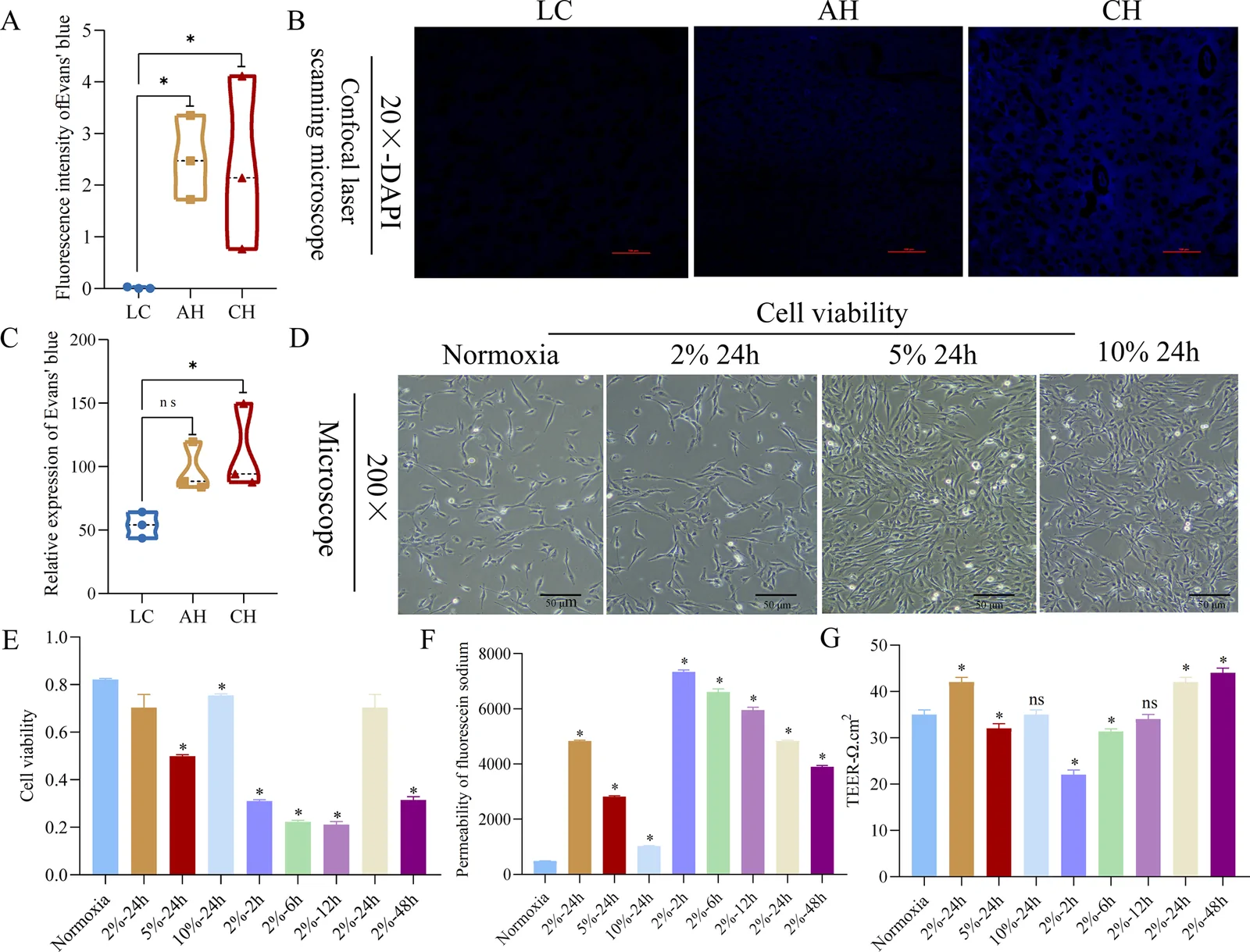
Variations in the permeability of the blood-brain barrier in rats and in vitro hCMEC/D_3_ under hypoxia. Note: **A**, **B**: results of confocal laser scanning microscopy. **C**: content of evans blue. **D**, **E**: the survival status of hCMEC/D_3_ cell. **F**, **G**: determination of hCMEC/D_3_ cell permeability and integrity. LC, AH, CH refer to the low-altitude control group, high-altitude acute hypoxia group, and high-altitude chronic hypoxia group, respectively. hCMEC/D_3_ were cultured at 2% (2%-24 h), 5% (5%-24 h), 10% (10%-24 h) oxygen concentrations for 24 h, and at 2% oxygen concentrations for 2 h (2%-2 h), 6 h (2%-6 h), 12 h (2%-12 h), 24 h (2%-24 h), and 48 h (2%-48 h), respectively, (*n* = 3, ^*^p < 0.05 compared to the low-altitude control group/normoxia group)

Evans blue staining and confocal laser scanning microscopy experiments for assessing blood-brain barrier alterations under hypoxia. Rat tails, eyeballs, thoraxes, and brain tissues all displayed a blue coloration following Evans blue injection into the rat tail vein. The intensity of Evans blue staining of the blood-brain barrier was increased in the high-altitude acute hypoxia group and the chronic hypoxia group of rats, as shown in Fig. 3A. By quantifying the fluorescence intensity, we observed significantly high values in both the acute and chronic high-altitude hypoxia groups. (Figs. 3B and 3C).

As shown in Fig. 3D, cultured hCMEC/D_3_ exhibited low granularity, and were transparent, and well-outlined. As shown in Fig. 3E, cell viability varied under various hypoxic conditions and durations, decreasing by half under treatment with 2% 2 h hypoxic treatment. Sodium fluorescein revealed that hCMEC/D_3_ treated with varying hypoxia doses and periods had enhanced permeability, with fluorescent intensity being significantly higher in the hypoxia group as compared to the normoxic group, treatment modalities included 2% hypoxia for 24 h, 10% hypoxia for 24 h, 5% hypoxia for 24 h, 2% hypoxia for 2 h, 2% hypoxia for 6 h, 2% hypoxia for 12 h, 2% hypoxia for 24 h, and 2% hypoxia for 48 h, with the 2% for 2 h treatment inducing the highest fluorescence intensities (Fig. 3F). Analysis of trans-endothelial resistance revealed that hCMEC/D_3_ cell integrity and permeability were affected by treatments based on varying hypoxia durations and concentrations (Fig. 3G). As compared to the normoxic treatment, treatment with hypoxia at 2% for 24 h and at 2% for 48 h induced significantly higher resistance, while treatment with hypoxia at 5% for 24 h, at 2% for 2 h, and at 2% for 6 h induced significantly lower resistance, with the 2% for 2 h treatment inducing the lowest resistance value, with high permeability.

### DIA proteomics

#### Normalization of protein quantification and protein sequencing

**Fig. 4:**
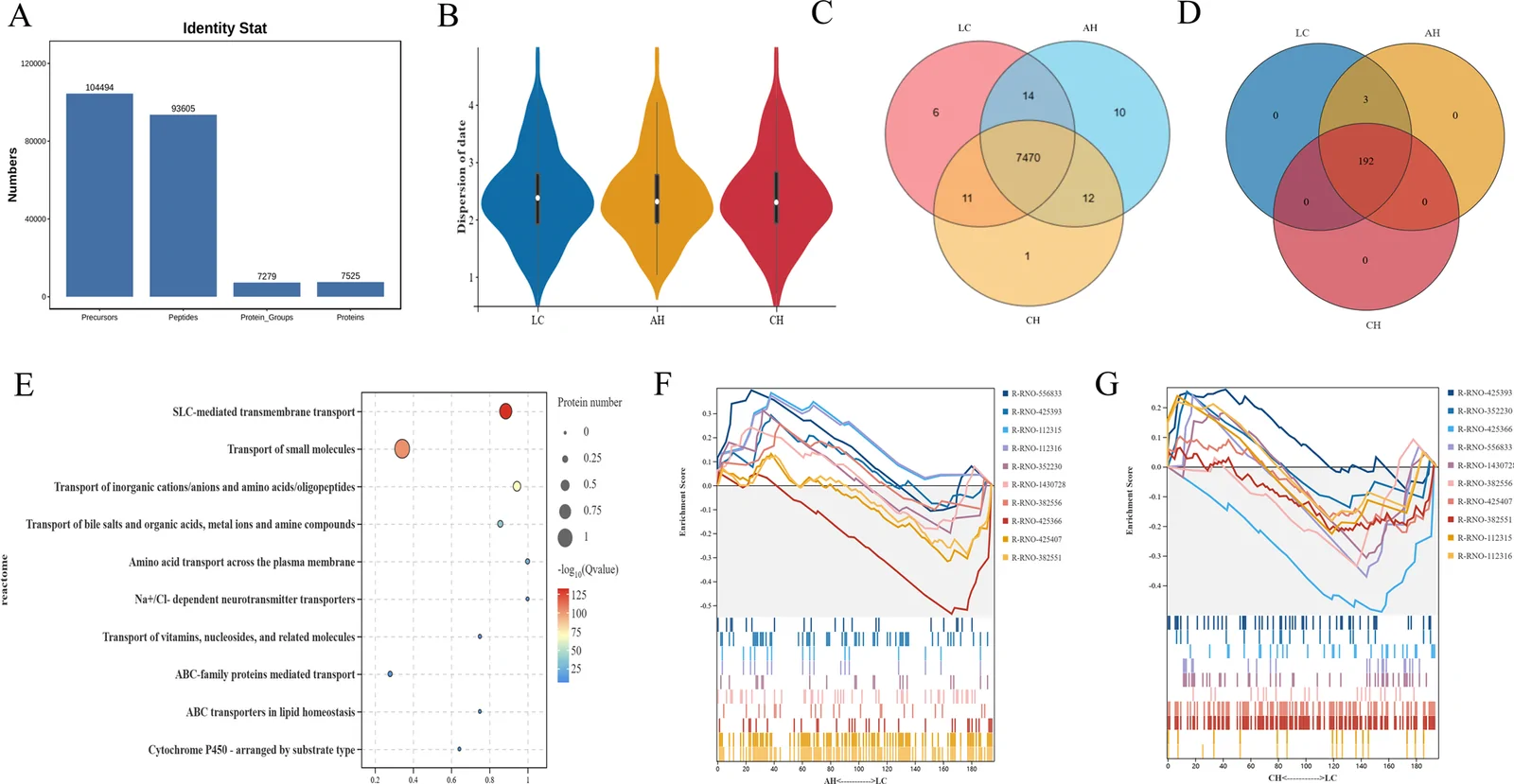
DIA sequencing analysis of rat brains under high-altitude hypoxia. Note: **A**, **B**: normalisation and sequencing results for groups of samples. **C**, **D**: differential genes in each group of rats. **E**: reactome enrichment, **F**, **G**: GSEA analysis of target proteins. LC, AH, CH refer to the low-altitude control group, high-altitude acute hypoxia group, and high-altitude chronic hypoxia group, respectively. (*n* = 3, ^*^p < 0.05 compared to the low-altitude control group)

Although samples are usually pre-measured before being injected into the mass spectrometer, they must be normalized to the intensity of spectral peaks to prevent the appearance of signals with significant background differences. Figure 4A shows the distribution of peptide quantification values for each group following normalization. Most samples exhibited approximately the same signal strength, fulfilling the requirements for further testing. After all the samples were filtered via quality control, 104,494 spectra and 7,279 proteomes were obtained, with the number of peptides and proteins being 93,605 and 7,525, respectively (Fig. 4B). Quantitative analysis of protein counts in the three groups revealed the average quantitative protein values per sample in the low-altitude control, high-altitude acute hypoxia, and high-altitude chronic hypoxia groups to be 1,605, 1,672, and 1,664, respectively.

#### Quantitative analysis of blood-brain barrier proteins in rats under high-altitude hypoxia

We filtered out a total of 179 drug transporters, 16 drug-metabolizing enzymes and 8 tight-junction proteins were identified, mainly Slc25a4, Slc1a2, Slc6a11, Slc17a7, Slc3a2, Slc2a1, Slc32a1, Abcb1, Abcg2, Slco1a2 and CYP4F1, CYP46A1, CYP51, CYP7B1, CYP27A1, CYP2S1, CYP2D1, CYP2B1, CYP4×1, CYP3A1, *etc*, and Tjp1, Tjp2, Cldnd1, Cldn10, Cldn11, Cldn7, Cldn12 and Cldn3. Following qualitative analysis, the average peak areas of the top three MS1 peptides with an FDR of less than 1.0% were screened for major drug transporters, drug-metabolizing enzymes and tight-junction proteins. The results show that the mean concentrations of drug transporters were 1,893.60, 2,004.32, and 1,989.97 in the low-altitude control group, high-altitude acute hypoxia group, and high-altitude chronic hypoxia group, respectively; the mean concentrations of drug-metabolizing enzymes were 147.83, 175.88, and 168.80, respectively; and the mean concentrations of tight-junction proteins were 1,124.85, 706.43, and 912.92, respectively (Table 1).

**Table 1:** Protein quantification in the brain tissues of rats from three groups

Protein	Group	Sample 1 (U)	Sample 2 (U)	Sample 3 (U)	Mean ± SD
Drug transporters	LC	1980.30	1899.97	1800.54	1893.60 ± 73.53
	AH	2173.56	1934.43	1904.98	2004.32 ± 120.27
	CH	2035.70	1960.13	1974.07	1989.97 ± 32.84
Drug- metabolizing enzymes	LC	153.75	149.34	140.41	147.83 ± 5.55
	AH	161.21	158.48	207.96	175.88 ± 22.71
	CH	182.13	159.11	165.16	168.80 ± 9.75
Tight-junction proteins	LC	1178.13	1203.18	993.25	1124.85 ± 93.62
	AH	522.25	1042.20	554.84	706.43 ± 237.80
	CH	683.90	837.69	1217.16	912.92 ± 224.11

Note: LC, AH, CH refer to the low-altitude control group, high-altitude acute hypoxia group, and high-altitude chronic hypoxia group, respectively (*n* = 3, data are expressed as the mean ± SD values)

For the subsequent analyses, we selected key drug transporters Abcb1, Abcg2, Slco1a2 and drug-metabolizing enzymes CYP2D1, CYP2B1, CYP3A1, along with the tight junction protein Tjp1, all of which are implicated in drug disposition and barrier function Table 2 revealed that protein expression of Abcb1 and Slco1a2 was elevated, Abcg2 was reduced, drug-metabolizing enzyme CYP3A1 was elevated, CYP2B1 and CYP2D1 were reduced and Tjp1 was reduced in the blood-brain barrier of rats in the high-altitude acute and chronic hypoxia groups when compared with the low-altitude control group.

**Table 2:** Expression of drug transporters and drug-metabolizing enzymes and tight-junction at the blood-brain barrier in three groups of rats under high-altitude hypoxia

Protein	LC (U)	AH (U)	CH (U)
Abcb1	510.07 ± 22.79	550.20 ± 82.15	635.57 ± 25.69^*^
Abcg2	121.41 ± 18.84	100.52 ± 16.59	75.86 ± 13.65^*^
Slco1a2	103.02 ± 43.16	123.16 ± 21.65	122.33 ± 35.56
CYP3A1	15.17 ± 2.43	16.59 ± 7.73	32.22 ± 11.89^*^
CYP2B1	160.02 ± 24.88	105.84 ± 8.99^*^	105.11 ± 19.56^*^
CYP2D1	167.64 ± 30.36	125.77 ± 7.84^*^	116.58 ± 15.08^*^
Tjp1	622.69 ± 22.02	521.35 ± 43.44^*^	546.05 ± 58.48

Note: LC, AH, CH refer to the low-altitude control group, high-altitude acute hypoxia group, and high-altitude chronic hypoxia group, respectively (Data are expressed as the mean ± SD values. *n* = 3, ^*^p < 0.05 compared to the low-altitude control group)

#### Analysis of the differentially expressed proteins at the blood-brain barrier

Based on the results of protein characterisation, the blood-brain barrier differential proteins were identified, Fig. 4C showed that there were 7,479 common genes among the low-altitude control group, high-altitude acute and chronic hypoxia groups, with 5, 7, and 1 specific genes. Sag, Cbln3, Pmp2, Gpr151, and Myh8 were the specific genes in the low-altitude control group; stomatin, Cldn7, Klc3, Nme5, Frmd7, Ap1m2, and Sp8 were in the high-altitude acute hypoxia group; and Igfals were in the high-altitude chronic hypoxia group. Subsequently we performed a differential analysis of the drug transporters and metabolizing enzymes from the shared genes mentioned above and found that the target proteins shared 192 genes. Three genes, Slc44a4, Slc2a4, and Slc39a12, were shared by the high-altitude acute hypoxia group and the low-altitude control group, which were in agreement with the findings of the quantitative analysis of the proteins (Fig. 4D), detailed quantitative results are presented in Supplementary Material S3 and S4.

#### Reactome enrichment and GSEA analysis of target proteins in brain tissues under high-altitude hypoxia

Graphing of Reactome-enriched bubble plots revealed that the pathways enriched across the three rat groups were primarily associated with SLC-mediated transmembrane transport, small molecule transport, inorganic cation/anion transport, amino acid and oligopeptide transport, bile salt and organic acid transport, metal ion and amine compound transport, amino acid transport across the plasma membrane, Na^+^/Cl^−^ -dependent neurotransmitter transporters, vitamin, nucleoside, and related molecule transport, ABC family protein-mediated transport, ABC transporters involved in lipid homeostasis, and cytochrome P450 metabolism organized by substrate type (Fig. 4E). While Reactome enrichment analysis effectively highlighted common pathways upregulated in the brain tissues of all three groups, they did not clarify pathways that may differ between the groups.

Reactome enrichment analysis’s regulatory effects can be more fully explained by GSEA analysis, which effectively compensates for the limited data mining capacity of traditional enrichment analyses for effective information on microeffective genes. Through clustering GSEA-reactome functional enrichment analyses, we identified variations in the gene function prediction of drug transporters and metabolizing enzymes of the blood-brain barrier across the rat groups. Compared to the low-altitude control group, the main enriched pathways related to drug metabolism in the blood-brain barrier of rats in the high-altitude acute hypoxia group were: amino acid transport across the plasma membrane, transmission across chemical synapses, metabolism of lipids, transport of inorganic cations/anions and amino acids/oligopeptides, and neural system (Fig. 4F). The high-altitude chronic hypoxia group were: transport of inorganic cations/anions and amino acids/oligopeptides, amino acid transport across the plasma membrane, transport of bile salts and organic acids, metal salts and organic acids, metal ions and amine compounds, metabolism of lipids and metabolism (Fig. 4G). Furthermore, our analyses revealed that the functions of genes involved in target proteins were also altered in high-altitude hypoxia, with the main altered being Slc1a5, Slc17a7, Abcg2, Abcd1, Slco1a4, and CYP27A1, CYP4F1, CYP46A1, CYP2D4, CYP4B1 and others, these changes in protein expression may lead to modifications in the permeability of the blood-brain barrier, which in turn impacts drug transport. This suggests that modifications to the metabolic pathways mediated by drug transporters and metabolizing enzymes in the blood-brain barrier in the hypoxic environment of the plateau may be intimately linked to the management of disorders of the central nervous system.

### Expression of major drug transporters, metabolic enzymes and tight-junction proteins in the blood-brain barrier and hCMEC/D_3_ under hypoxia

**Fig. 5:**
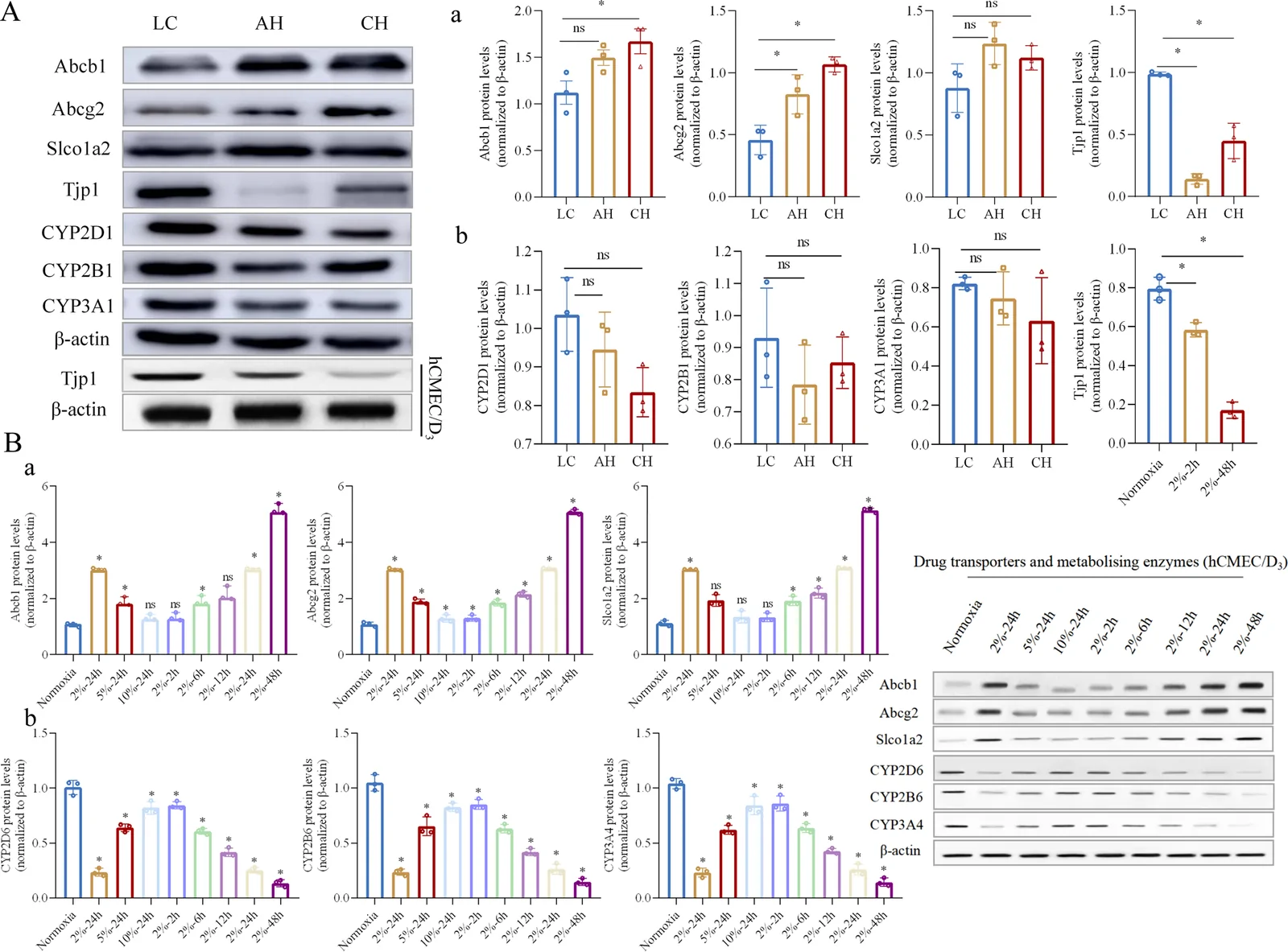
Protein expression of drug transporters, drug-metabolizing enzymes and tight-junction in blood-brain barrier and hCMEC/D_3_ cell under high-altitude hypoxia. Note: **A**(a): protein expression of major drug transporters and Tjp1 in blood-brain barrier. **A**(b): protein expression of major drug-metabolizing enzymes and Tjp1 at the cellular level. **B**(a): protein expression of major drug transporters in hCMEC/D_3_. **B**(b): protein expression of major drug-metabolizing enzymes in hCMEC/D_3_ cell. LC, AH, CH refer to the low-altitude control group, high-altitude acute hypoxia group, and high-altitude chronic hypoxia group, respectively. (*n* = 3, ^*^p < 0.05 compared to the low-altitude control group)

As shown in Fig. 5A, the protein expression of Abcg2, a drug transporter of the blood-brain barrier, was significantly elevated in the high-altitude acute hypoxia group of rats, protein expression of Abcb1 and Abcg2 was significantly elevated in the high-altitude chronic hypoxia group, compared with the low-altitude control group. Compared with the low-altitude control group, the protein expression of Abcb1 in the high-altitude acute hypoxia group showed an increasing trend, and the protein expression of Slco1a2 in both the high-altitude acute and chronic hypoxia groups also exhibited a similar trend; however, none of these differences reached statistical significance. The expression of the blood-brain barrier drug-metabolizing enzymes CYP2D1, CYP2B1 and CYP3A1 tended to be reduced in the high-altitude acute and chronic hypoxia group of rats when compared to the low-altitude control group, but none of these differences reached statistical significance. And Tjp1 protein expression was significantly reduced in the blood-brain barrier and hCMEC/D_3_ when compared to low-altitude control group.

As shown in Fig. 5B, in hCMEC/D_3_, compared with the normoxia group, the protein expression of Abcb1 and Slco1a2 showed an increasing trend after 2% hypoxia exposure for 2 hours, but without statistical significance. However, after 2% hypoxia exposure for 48 hours, the protein expression of Abcb1 was significantly elevated, and the protein expression of Slco1a2 was also significantly increased. The protein expression of Abcg2 was significantly elevated after both 2 hours and 48 hours of 2% hypoxia exposure. Compared with the normoxic group, the protein levels of drug-metabolizing enzymes CYP2D6, CYP2B6 and CYP3A4 were significantly reduced after hypoxia of 2% 2 h and 2% 48 h in hCMEC/D_3_. Overall, the hypoxic environment elevated the levels of protein expression of the drug transporters Abcb1, Abcg2, and Slco1a2, and reduced the levels of drug-metabolizing enzymes CYP2D6, CYP2B6, and CYP3A4, and reduced the levels of Tjp1.

### mRNA expression of major drug transporters, metabolic enzymes and tight-junction proteins at the blood-brain barrier and hCMEC/D_3_ under hypoxia

**Fig. 6:**
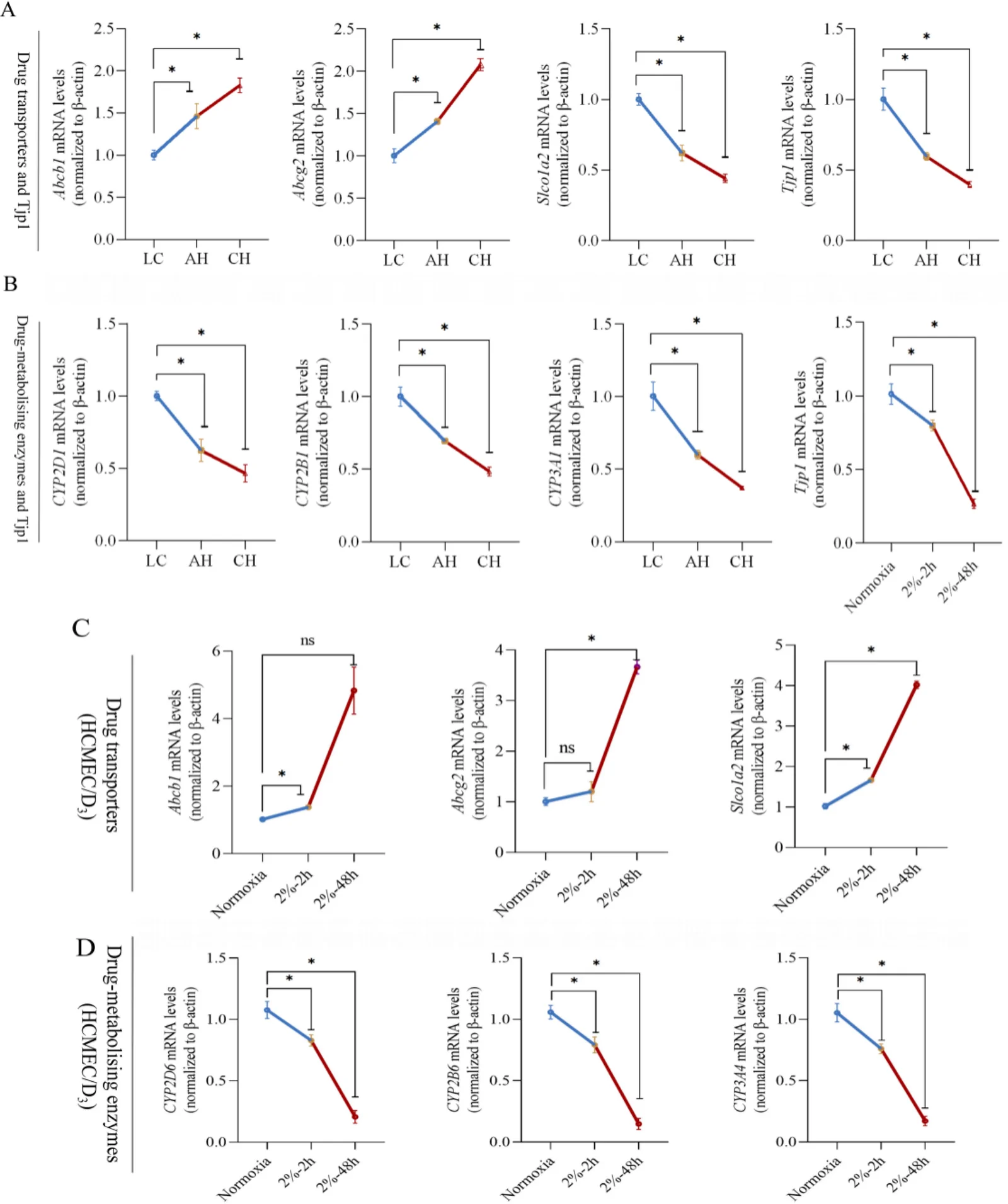
mRNA expression of drug transporters, drug-metabolizing enzymes and tight-junction proteins at the blood-brain barrier and hCMEC/D_3_ cell under high-altitude hypoxia. Note: **A**: mRNA expression of major drug transporters and *Tjp1* at the blood-brain barrier. **B**: mRNA expression of major drug-metabolizing enzymes and *Tjp1* at the cellular level. **C**: mRNA expression of major drug transporters in hCMEC/D_3_. **D**: mRNA expression of major drug-metabolizing enzymes in hCMEC/D_3_ cell. LC, AH, CH refer to the low-altitude control group, high-altitude acute hypoxia group, and high-altitude chronic hypoxia group, respectively. (*n* = 3, ^*^p < 0.05 compared to the low-altitude control group)

In comparison with the low-altitude control group, mRNA expression of the blood-brain barrier drug transporters *Abcb1* and *Abcg2* was significantly elevated, and mRNA expression of *Slco1a2* was significantly reduced in the high-altitude acute and chronic hypoxia group of rats (Fig. 6A). In comparison with the low-altitude control group, mRNA expression of the drug-metabolizing enzymes *CYP2D1*, *CYP2B1* and *CYP3A1* was significantly reduced (Fig. 6B). In comparison with low-altitude control group, mRNA expression of *Tjp1* was significantly reduced in the blood-brain barrier and hCMEC/D_3_, as show in Figs. 6A and 6B.

Compared with the normoxic group, in hCMEC/D_3_ exposed to 2% hypoxia, the mRNA level of Abcb1 was significantly upregulated at 2 hours, but the increase at 48 hours was not statistically significant. The mRNA expression of Abcg2 was significantly increased at 48 hours, while the elevation at 2 hours showed a trend without significance. The mRNA expression of Slco1a2 was significantly elevated at both 2 hours and 48 hours (Fig. 6C). The mRNA levels of drug-metabolizing enzymes *CYP2D6*, *CYP2B6* and *CYP3A4* were significantly reduced after 2% 2 h and 2% 48 h of hypoxia (Fig. 6D). The hypoxic environment induced an increase in the mRNA expression levels of the drug transporters, *Abcb1* and *Abcg2*, and reduced the mRNA expression levels of drug-metabolism and tight-junction proteins, *CYP2D6*, *CYP2B6*, *CYP3A4*, and *Tjp1*.

### Methodological validation

Method selectivity was evaluated by analyzing blank plasma, blank plasma spiked with an internal standard (IS), and blank plasma spiked with both the IS and the target drug. Supporting material Fig. S1 illustrates that the method has demonstrated good specificity for hydrochloride propranolol, hydrochloride verapamil, zolmitriptan and hydrochloride mexiletine, ubenimex, and sumatriptan succinate, and that endogenous substances in plasma and brain tissues did not interfere with the chromatographic analysis and quantitative requirements were satisfied.

In the linear regression model, drug concentrations in quality control samples were used as horizontal coordinates and the peak area was used as the vertical coordinate. The linear equations for propranolol hydrochloride in plasma and brain tissue were *Y* = 0.0203*X* + 0.0218 (R^2^ = 0.9985, linear range: 1.17 - 600.00 ng/mL) and *Y* = 0.0264*X* + 0.3721 (R^2^ = 0.9931, linear range: 1.17 - 600.00 ng/mL). Linear equations for verapamil hydrochloride in plasma and brain tissues were *Y* = 0.0035*X* + 0.0314 (R^2^ = 0.9987, linear range: 1.17 - 300.00 ng/mL) and *Y* = 0.005*X* + 0.0002 (R^2^ = 0.9998, linear range: 0.07 - 37.50 ng/mL). Linear equations of zolmitriptan were *Y* = 0.0007*X* + 0.0032, (R^2^ = 0.9992, linear range: 1.17 - 600.00 ng/mL) and *Y* = 0.0016*X* + 0.0003 (R^2^ = 0.9993, linear range: 0.07 - 37.50 ng/mL), which demonstrated good linearity of the substance to be measured over the set concentration range.

As shown in supporting materials S6–S14, precision and accuracy, stability experiments, and matrix effects all complied with the requirements for biological sample testing.

### Effects of high-altitude hypoxia on drug pharmacokinetics in plasma and brain tissues

**Fig. 7:**
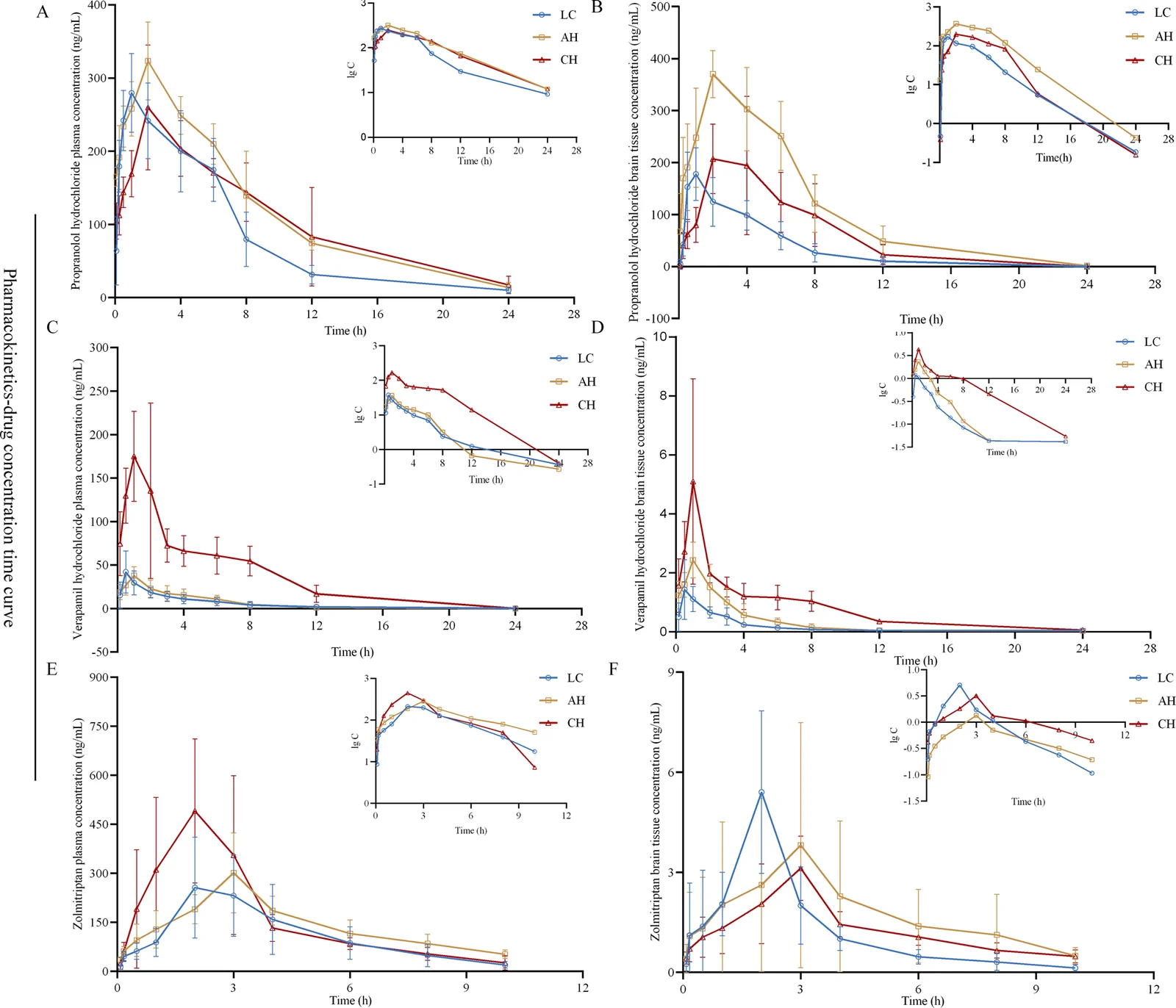
Mean plasma concentration and brain tissue concentration-time curves of drug for rats after exposure to high-altitude hypoxia (*n* = 6). Note: **A**-**B**: mean drug concentration time curves in plasma and brain tissue for propranolol hydrochloride, **C**-**D**: mean drug concentration time curves of verapamil hydrochloride in plasma and brain tissue, **E**-**F**: mean drug concentration time curves of zolmitriptan in plasma and brain tissue. LC, AH, CH refer to the low-altitude control group, high-altitude acute hypoxia group, and high-altitude chronic hypoxia group, respectively

The average concentration-time curves for plasma and brain tissue drugs of propranolol hydrochloride, verapamil hydrochloride and zolmitriptan administered orally to rats in the low-altitude control group, in the high-altitude acute and chronic hypoxia groups are depicted in Fig. 7. The relevant pharmacokinetic parameters are described in Tables 3, 4, 5, respectively. Compared with the low-altitude control group of propranolol hydrochloride, plasma AUC_0–24_, AUC_0-∞_, MRT_0–24_ and *T*_max_ were significantly elevated, CL/F was significantly reduced, MRT_0-∞_, *t*_1/2_ and *C*_max_ tended to be elevated, and *K*_e_ and *V*_d_/F tended to reduced in the high-altitude acute hypoxia group group; the plasma of the chronic hypoxia group had significantly elevated *T*_max_, significantly reduced CL/F, a trend towards elevated AUC_0–24_, AUC_0-∞_, MRT_0–24_, MRT_0-∞_, *t*_1/2_, and a trend towards reduced *C*_max_ and *K*_e_. In the brain tissue of the high-altitude acute hypoxia group, AUC-brain_0–24_, AUC-brain_0-∞_, MRT-brain_0–24_, MRT-brain_0-∞_, *T*_max_-brain and *C*_max_-brain were significantly elevated, CL/F-brain and *V*_d_/F-brain was significantly reduced, *t*_1/2_-brain showed an elevated trend, *K*_e_-brain showed a reduced trend; and in the brain tissue of the chronic hypoxia group, MRT_0–24_-brain, MRT_0-∞_-brain and *T*_max_-brain were significantly elevated, CL/F-brain was significantly reduced, AUC_0–24_-brain, AUC_0-∞_-brain, *t*_1/2_-brain and *C*_max_-brain showed an elevated trend, *K*_e_-brain and *V*_d_/F-brain showed a reducing trend (Table 3).

**Table 3:** Main pharmacokinetic parameters of propranolol hydrochloride in the plasma and brain tissues under high-altitude hypoxia

Parameter	Plasma			Brain		
	LC	AH	CH	LC	AH	CH
AUC_0–24_ (ng/mL·h)	2011.61 ± 389.03	2844.23 ± 314.77^*^	2561.92 ± 671.87	799.25 ± 134.20	2489.76 ± 832.68^*^	1358.72 ± 667.55
AUC_0-∞_ (ng/mL·h)	2038.05 ± 416.98	2919.02 ± 318.13^*^	2654.40 ± 731.85	871.64 ± 149.15	2737.03 ± 777.34^*^	1527.73 ± 658.12
MRT_0–24_ (h)	5.50 ± 0.57	6.37 ± 0.22^*^	7.05 ± 1.12	3.58 ± 0.66	4.55 ± 0.74^*^	4.51 ± 0.60^*^
MRT_0-∞_ (h)	6.10 ± 0.77	7.13 ± 0.60	8.13 ± 1.59	4.45 ± 1.42	5.73 ± 0.88^*^	5.82 ± 0.34^*^
*t*_1/2_ (h)	3.34 ± 0.85	4.29 ± 0.75	4.43 ± 1.60	2.72 ± 1.14	3.34 ± 0.78	3.12 ± 0.64
*K*_e_ (h^−1^)	0.22 ± 0.06	0.17 ± 0.03	0.18 ± 0.07	0.29 ± 0.10	0.22 ± 0.05	0.23 ± 0.03
*T*_max_ (h)	1.08 ± 0.49	1.83 ± 0.41^*^	2.67 ± 1.03^*^	0.92 ± 0.20	2.33 ± 0.82^*^	2.33 ± 0.82^*^
CL/F (L/h/kg)	0.010 ± 0.00	0.007 ± 0.00^*^	0.008 ± 0.00^*^	0.024 ± 0.01	0.008 ± 0.00^*^	0.016 ± 0.01^*^
*V*_d_/F (L/kg)	0.05 ± 0.01	0.04 ± 0.01	0.05 ± 0.02	0.09 ± 0.04	0.04 ± 0.01^*^	0.07 ± 0.03
*C*_max_ (ng/mL)	284.21 ± 55.52	324.22 ± 53.11	280.57 ± 69.40	206.27 ± 34.24	371.70 ± 47.56^*^	245.10 ± 122.68

Note: Data are expressed as the mean ± SD values (*n* = 6). ^*^p < 0.05 compared to the low-altitude control group. AUC: area under the concentration time curve, MRT: mean residence time, *t*_1/2_: half-life, *T*_max_: peak time, *K*_e_: elimination rate, CL/F: total plasma/brain clearance, *V*_d_/F: apparent volume of distribution, *C*_max_: the maximum samples concentration. LC, AH, CH refer to the low-altitude control group, high-altitude acute hypoxia group, and high-altitude chronic hypoxia group, respectively

**Table 4:** Main pharmacokinetic parameters of verapamil hydrochloride in the plasma and brain tissues under high-altitude hypoxia

Parameter	Plasma			Brain		
	LC	AH	CH	LC	AH	CH
AUC_0–24_ (ng/mL·h)	128.67 ± 49.44	137.04 ± 56.25	834.85 ± 219.40^*^	4.29 ± 1.09	7.98 ± 2.22^*^	19.05 ± 3.18^*^
AUC_0-∞_ (ng/mL·h)	147.92 ± 65.54	181.77 ± 70.04	965.85 ± 228.63^*^	4.31 ± 1.11	8.21 ± 2.30^*^	19.43 ± 3.19^*^
MRT_0–24_ (h)	3.50 ± 1.63	2.96 ± 0.41	4.20 ± 0.46	4.73 ± 0.87	3.72 ± 0.37	5.20 ± 1.16
MRT_0-∞_ (h)	4.63 ± 1.90	5.57 ± 2.23	6.28 ± 2.41	6.19 ± 1.24	4.72 ± 0.48^*^	5.70 ± 1.11
*t*_1/2_ (h)	3.79 ± 1.29	4.21 ± 1.70	4.47 ± 1.92	3.40 ± 0.22	4.68 ± 1.31	4.27 ± 0.55
*K*_e_ (h^−1^)	0.20 ± 0.07	0.18 ± 0.05	0.18 ± 0.07	0.20 ± 0.01	0.16 ± 0.05	0.16 ± 0.02
*T*_max_ (h)	0.58 ± 0.20	1.00 ± 0.00^*^	1.17 ± 0.41^*^	0.58 ± 0.20	1.17 ± 0.41^*^	1.17 ± 0.41^*^
CL/F (L/h/kg)	0.16 ± 0.07	0.13 ± 0.05	0.02 ± 0.01^*^	4.88 ± 1.17	2.59 ± 0.69^*^	1.05 ± 0.18^*^
*V*_d_/F (L/kg)	0.82 ± 0.35	0.73 ± 0.31	0.14 ± 0.05^*^	24.04 ± 6.26	16.88 ± 4.35	6.49 ± 1.28^*^
*C*_max_ (ng/mL)	42.80 ± 23.63	37.64 ± 10.25	206.66 ± 80.54^*^	1.57 ± 0.94	2.49 ± 0.64	5.19 ± 3.38^*^

Note: Data are expressed as the mean ± SD values (*n* = 6). ^*^p < 0.05 compared to the low-altitude control group. AUC: area under the concentration time curve, MRT: mean residence time, *t*_1/2_: half-life, *T*_max_: peak time, *K*_e_: elimination rate, CL/F: total plasma/brain clearance, *V*_d_/F: apparent volume of distribution, *C*_max_: the maximum samples concentration. LC, AH, CH refer to the low-altitude control group, high-altitude acute hypoxia group, and high-altitude chronic hypoxia group, respectively

**Table 5:** Main pharmacokinetic parameters of zolmitriptan in the plasma and brain tissues under high-altitude hypoxia

Parameter	Plasma			Brain		
	LC	AH	CH	LC	AH	CH
AUC_0–24_ (ng/mL·h)	1115.43 ± 494.30	1374.42 ± 164.73	1661.68 ± 442.44^*^	13.57 ± 5.30	19.39 ± 19.79	14.84 ± 8.39
AUC_0-∞_ (ng/mL·h)	1199.07 ± 532.56	1573.20 ± 219.34	1835.30 ± 487.79^*^	14.31 ± 5.83	21.62 ± 19.88	17.97 ± 10.11
MRT_0–24_ (h)	3.73 ± 0.43	4.18 ± 0.30	3.19 ± 0.63	2.80 ± 0.29	4.12 ± 0.43^*^	3.98 ± 0.40^*^
MRT_0-∞_ (h)	4.30 ± 0.55	5.63 ± 0.55^*^	4.23 ± 1.12	3.15 ± 0.40	5.84 ± 1.30^*^	6.22 ± 2.62^*^
*t*_1/2_ (h)	2.33 ± 0.38	2.87 ± 0.45	2.98 ± 1.19	2.48 ± 0.74	3.30 ± 0.87	4.02 ± 3.40
*K*_e_ (h^−1^)	0.30 ± 0.04	0.25 ± 0.04	0.27 ± 0.10	0.30 ± 0.08	0.23 ± 0.08	0.27 ± 0.13
*T*_max_ (h)	2.17 ± 0.41	3.00 ± 0.63^*^	2.50 ± 0.55	2.17 ± 0.41	3.17 ± 0.41^*^	3.17 ± 0.41^*^
CL/F (L/h/kg)	0.02 ± 0.01	0.01 ± 0.00	0.01 ± 0.00	1.59 ± 0.61	2.00 ± 1.98	1.33 ± 0.48
*V*_d_/F (L/kg)	0.07 ± 0.05	0.05 ± 0.01	0.05 ± 0.02	5.41 ± 2.00	10.75 ± 11.36	6.46 ± 4.66
*C*_max_ (ng/mL)	305.11 ± 147.20	319.51 ± 106.18	579.13 ± 208.30^*^	5.91 ± 2.73	5.49 ± 6.44	5.14 ± 5.61

Note: Data are expressed as the mean ± SD values (*n* = 6). ^*^p < 0.05 compared to the low-altitude control group. AUC: area under the concentration time curve, MRT: mean residence time, *t*_1/2_: half-life, *T*_max_: peak time, *K*_e_: elimination rate, CL/F: total plasma/brain clearance, *V*_d_/F: apparent volume of distribution, *C*_max_: the maximum samples concentration. LC, AH, CH refer to the low-altitude control group, high-altitude acute hypoxia group, and high-altitude chronic hypoxia group, respectively

Compared with the low-altitude control group of verapamil hydrochloride, plasma in the high-altitude acute hypoxia group had a significantly elevated T_max_, a tendency for elevated AUC_0–24_, AUC_0-∞_, MRT_0-∞_ and *t*_1/2_, a tendency for reduced MRT_0–24_, *K*_e_, CL/F, *V*_d_/F and *C*_max_; In the plasma of the high-altitude chronic hypoxia group, AUC_0–24_, AUC_0-∞_, *T*_max_ and *C*_max_ were significantly elevated, CL/F and *V*_d_/F were significantly reduced, and MRT_0–24_, MRT_0-∞_, and *t*_1/2_ tended to be elevated, while *K*_e_ tended to be reduced. In the brain tissue of the high-altitude acute hypoxia group, AUC-brain_0–24_, AUC-brain_0-∞_ and *T*_max_-brain were significantly elevated, MRT-brain_0-∞_ and CL/F-brain were significantly reduced, *t*_1/2_-brain and *C*_max_-brain showed an elevated trend, MRT-brain_0–24_, *K*_e_-brain and *V*_d_/F-brain showed a reduced trend; In brain tissues of the chronic hypoxia group, AUC-brain_0–24_, AUC-brain_0-∞_, *T*_max_-brain and *C*_max_-brain were significantly elevated, CL/F-brain and *V*_d_/F-brain were significantly reduced, its *t*_1/2_-brain and MRT-brain_0–24_ tended to be elevated, and MRT-brain_0-∞4_ and *K*_e_-brain tended to be reduced (Table 4).

Compared to zolmitriptan low-altitude control group, the plasma MRT_0-∞_ and *T*_max_ were significantly elevated, AUC_0–10_, AUC_0-∞_, MRT_0–10_, *t*_1/2_ and *C*_max_ tended to be elevated, *K*_e_, CL/F and *V*_d_/F trended to be reduced in the high-altitude acute hypoxia groups; the plasma AUC_0–10_, AUC_0-∞_ and *C*_max_ were significantly elevated, *t*_1/2_ and *T*_max_ tended to be elevated, and MRT_0–10_, MRT_0-∞_, *K*_e_, CL/F and *V*_d_/F trended to be reduced in the high-altitude chronic hypoxia groups. The brain tissue of the high-altitude acute and chronic hypoxia group had significantly elevated in MRT-brain_0–10_, MRT-brain_0-∞_ and *T*_max_-brain, with an elevated trend in AUC-brain_0–10_, AUC-brain_0-∞_, *t*_1/2_-brain and *V*_d_/F-brain, and a reduced trend in *K*_e_-brain and *C*_max_-brain (Table 5). Therefore, in vivo metabolism of propranolol hydrochloride, verapamil hydrochloride, and zolmitriptan was slowed due to high-altitude hypoxia, and this was mostly evidenced by a prolonged *t*_1/2_, decreased CL/F, and elevated AUC_0-t_.

### Impact of high-altitude hypoxia on the brain distribution of propranolol hydrochloride, verapamil hydrochloride and zolmitriptan in the brain

**Fig. 8:**
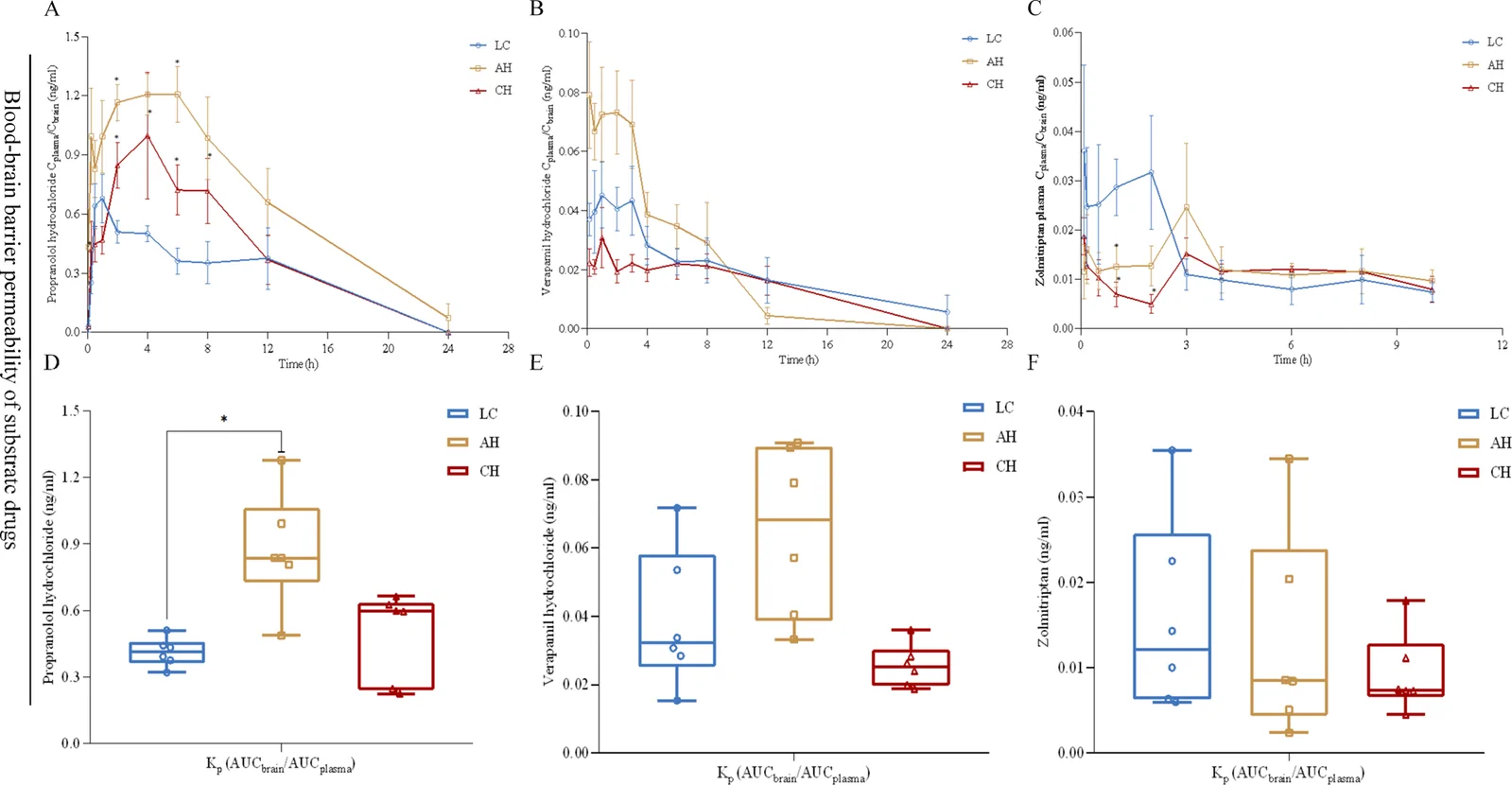
Effect of high-altitude hypoxia on drug transport across the blood-brain barrier (*n* = 6, ^*^p < 0.05 compared to the low-altitude control group). Note: **A**-**C**: blood-brain barrier drug concentrations of propranolol hydrochloride, verapamil hydrochloride, and zolmitriptan, **D**-**F**: brain-blood distribution ratio of propranolol hydrochloride, verapamil hydrochloride and zolmitriptan, LC, AH, CH refer to the low-altitude control group, high-altitude acute hypoxia group, and high-altitude chronic hypoxia group, respectively

As compared to the low-altitude propranolol hydrochloride control group, the *C*_brain_/*C*_plasma_ in the high-altitude acute hypoxia group was significantly elevated at 2 and 6 h, with an elevated trend at 0.25, 0.5, 1, 4, 8, 12, and 24 h; in the high-altitude chronic hypoxia group, the *C*_brain_/*C*_plasma_ was significantly elevated at 0.25, 2, 4, and 6 h (Fig. 8A). As compared to the low-altitude control group of verapamil hydrochloride, Fig. 8B shows that the *C*_brain_/*C*_plasma_ in the high-altitude acute hypoxia group tended to be elevated at 0.17, 0.5, 1, 2, 3, 4, 6, and 8 h, and reduced at 12 and 24 h; in the high-altitude chronic hypoxia group, they tended to be reduced at 0.17, 0.5, 1, 2, 3, 4, 6, 8, 12, and 24 h. In comparison to the low-altitude control group of zolmitriptan, the *C*_brain_/*C*_plasma_ in the high-altitude acute hypoxia group was significantly reduced at 1 h, trended to reduced at 0.08, 0.17, 0.5, and 2 h, and trended to be elevated at 3, 4, 6, 8, and 10 h. In the high-altitude chronic hypoxia group, the *C*_brain_/*C*_plasma_ was significantly reduced at 1 h and 2 h, tended to reduce at 0.08, 0.17, and 0.5 h, and tended to elevate at 3, 4, 6, 8, and 10 h (Fig. 8C).

When compared to the propranolol hydrochloride low-altitude control group, Figure 8D shows that Kp_(AUCbrain/AUCplasma)_ was significantly elevated in the high-altitude acute hypoxia group and trended to be higher in the high-altitude chronic hypoxia group compared to the propranolol hydrochloride low-altitude control group. When compared with the low-altitude control group of verapamil hydrochloride, Kp_(AUCbrain/AUCplasma)_ tended to elevate in the high-altitude acute hypoxia group and to reduce in the high-altitude chronic hypoxia group (Fig. 8E). The high-altitude acute and chronic hypoxia groups had a trend toward reduced Kp_(AUCbrain/AUCplasma)_ compared with the zolmitriptan low-altitude control group (Fig. 8F). We found that relative propranolol hydrochloride exposure in brain tissues elevated, while zolmitriptan exposure reduced, under high-altitude hypoxia. This suggests that propranolol hydrochloride and zolmitriptan transport across the blood-brain barrier elevates and reduces, respectively, under high-altitude hypoxia.

### Variations in drug uptake and transport in hCMEC/D_3_ under hypoxia

#### Drug concentration screening

**Fig. 9:**
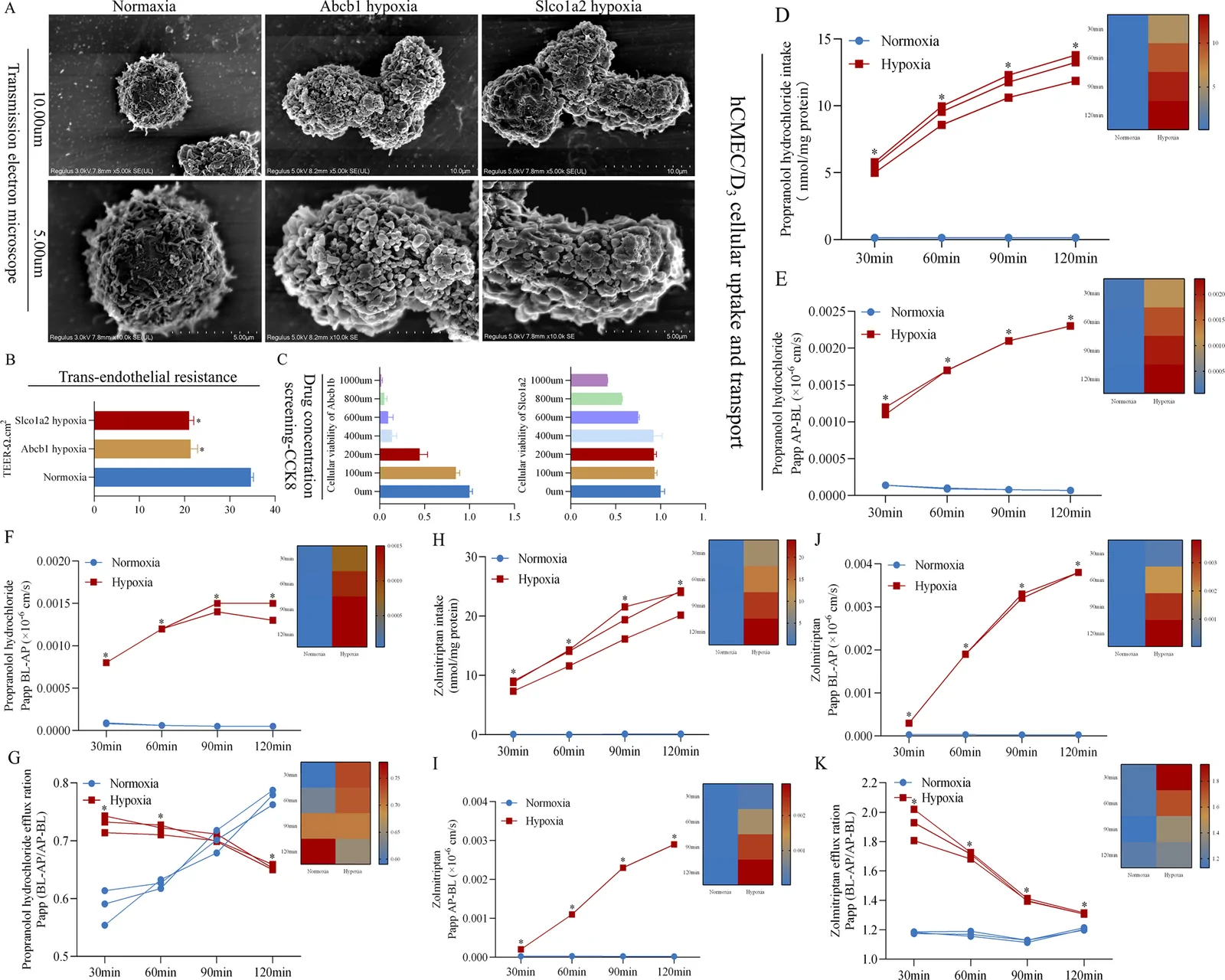
Variations in propranolol hydrochloride and zolmitriptan in drug uptake and transport in hCMEC/D_3_ under hypoxia. Note: **A**: results of hCMEC/D_3_ cellular transmission electron microscope. **B**: results of trans-endothelial resistance experiments. **C**: results of drug concentration screening of propranolol hydrochloride and zolmitriptan. **D**: intake of propranolol hydrochloride. **E**: propranolol hydrochloride transit rate (AP-BL). **F**: propranolol hydrochloride transit rate (BL-AP). **G**: propranolol hydrochloride efflux rate. **H**: zolmitriptan intake. **I**: zolmitriptan transit rate (AP-BL). **J**: zolmitriptan transit rate (BL-AP). **K**: zolmitriptan efflux rate. (*n* = 3, ^*^p < 0.05 compared to the normoxic group)

As shown in Fig. 9A, the established hCMEC/D_3_ single-cell layer model was observed to contain small particles, and to exhibit high transparency, distinct contours, and a good status. Transendothelial resistance findings demonstrated that the hCMEC/D_3_ single-cell layer model had well-defined density and integrity (Fig. 9B), which allowed for drug concentration screening and further experimentation. Using CCK8, the ideal drug concentrations in 0, 100, 200, 400, 600, 800, and 1000 μm were screened, and those for propranolol hydrochloride and zolmitriptan were found to be 100 and 400 μm, respectively (Fig. 9C), these values were used in subsequent experiments.

#### Effects of hypoxia on propranolol hydrochloride and zolmitriptan uptake and transport in hCMEC/D_3_

Congruent with the in vivo experiments, we examined the uptake and transport of propranolol hydrochloride and zolmitriptan in hCMEC/D_3_ to evaluate the function of Abcb1 and Slco1a2. Hypoxia significantly affected the uptake, rate of uptake, rate of efflux, and rate of efflux of propranolol hydrochloride and zolmitriptan, as shown in Fig. 9. Compared with the normoxic group, uptake was significantly elevated in the hypoxic group at 30, 60, 90, and 120 min after incubating the cells with propranolol hydrochloride for different times (Fig. 9D) (drug uptake assay). The uptake rate Papp AP-BL and efflux rate Papp BL-AP efflux rate were significantly elevated at 30, 60, 90 and 120 min, and the efflux rate was significantly elevated after 30 and 60 min of incubation and significantly reduced after 120 min (Fig.s. 9E-G) (drug translocation experiment).

Following drug exposure to the drug for varying durations, zolmitriptan uptake and translocation in the hypoxia group significantly elevated at 30, 60, 90, and 120 min relative to their values in the normoxic group (Fig. 9H). Consistent with this, both the Papp AP-BL and Papp BL-AP efflux rates significantly elevated at all evaluation time points (Figs. 9I, 9J). Moreover, the efflux rate was significantly high at 30, 60, 90, and 120 min under hypoxic conditions (Fig. 9K). The preceding results imply that the efflux of Abcb1 and the uptake of Slco1a2 were significantly elevated by hypoxia, it is implied that hypoxia increases blood-brain barrier transport of propranolol hydrochloride and decreases blood-brain barrier transport of zolmitriptan.

## Discussion

High-altitude hypoxia induced significant alterations in the brain-blood distribution ratio and the blood-brain barrier permeability coefficient, leading to either an increase or a decrease in the blood-brain barrier transport of propranolol hydrochloride, zolmitriptan, and verapamil hydrochloride, substrates of Abcb1, Slco1a2, and CYP2B1, respectively. These alterations correlated with hypoxia-mediated physiological changes, including enhanced blood brain barrier permeability, increased expression of efflux transporters (Abcb1 and Abcg2), reduced expression of the uptake transporter Slco1a2, and down-regulation of drug-metabolizing enzymes (CYP2D1, CYP2B1, and CYP3A1). The pharmacokinetic parameters of propranolol hydrochloride, zolmitriptan and verapamil hydrochloride in plasma and brain tissues were prolonged by *t*_1/2_, decreased by CL, and in vivo metabolism was significantly slowed down in the high-altitude hypoxia, suggesting that clinical use of the drug high altitude may be different from that in the plain area, and that the dosage of the administered drug should be carefully evaluated and adjusted. In high-altitude hypoxic environments, multiple factors including altitude, hypoxia, blood-brain barrier permeability, blood-brain barrier transport, drug transporters, and drug-metabolizing enzymes jointly regulate the absorption, distribution, and metabolism of central nervous system drugs. Further validation and investigation are needed to elucidate the specific molecular drivers governing drug transport across the blood-brain barrier.

We conducted HE staining and electron microscopic experiments to elucidate cell and tissue level barrier variations. In addition, we carried out Evans blue staining and sodium fluorescein-based in vivo and in vitro experiments to evaluate the effects of hypoxia on blood-brain barrier permeability. Under the high-altitude hypoxia environment, damage to the “filter” triggers a decrease in the expression of tight-junction proteins, an increase in the cellular and perivascular gaps, and the passage of different ions, molecules, and hazardous substances through the barrier, which lead to abnormalities in the structure and function of the barrier. It is consistent with the finding made by Halder and Milner that long-term moderate hypoxia causes fluorescein to flow beyond the cerebral vasculature, disrupting the blood-brain barrier [16]. In this study, the in vitro trend of increased permeability induced by hypoxia was largely consistent with the in vivo pattern of blood-brain barrier leakage in rats, indicating that this model can partially reflect the effects of hypoxia on blood-brain barrier function. Nevertheless, the in vitro monolayer model cannot fully capture the systemic factors involved in chronic hypoxic adaptation. Thus, the in vitro findings should be interpreted together with the in vivo data, and further validation using more physiological relevant models is needed in future studies.

According to our preliminary research, disruption to the blood-brain barrier may impact medication transport and therapy in the central nervous system via a variety of regulatory pathways, such as modifications to tight-junctions, drug transporters, drug-metabolizing enzymes, and pharmacokinetics [17, 18]. Transporters and metabolizing enzymes play a critical roles in drug transport and permeation. According to their function and location, the drug transporters in the brain can protect the central nervous system from exogenous toxins and promote or limit drug transport across the blood-brain barrier. In addition, the catalytic effects of drug metabolizing enzymes on exogenous substances, toxins and chemicals cannot be ignored in drug metabolism [10, 19]. In light of this, using DIA sequencing technology, we thoroughly evaluated drug transporters and metabolizing enzymes expressed in the blood-brain barrier during plateau hypoxia in a relatively thorough manner, and we have selected the drug transporters Abcb1, Abcg2, Slco1a2, which are more important for drug transport and metabolism in the blood-brain barrier, and the drug-metabolizing enzymes CYP2D1, CYP2B1 and CYP3A1 for mRNA and protein expression research, the specific trends of changes are presented in Supplementary Material S15. In accordance with the current investigation, Ozgür et al. [20] reported that hypoxia increased the expression of Abcb1, and that digoxin, the substrate drug, was more permeable in the blood-brain barrier. According to Cooper et al. [21], the Ivy GAP RNA-seq dataset may be linked to hypoxia-associated indicators and the increased expression of Slco1a2 in tumorous brain tissue. By establishing an in vitro blood-brain barrier model, Zolotoff et al. [22] found that chronic intermittent hypoxia for 6 h caused a structural and functional dysfunction of the blood-brain barrier, which in turn affected drug transport by upregulating Abcb1 expression and activity and downregulating Abcg2, this is consistent with the DIA sequencing results of this experiment. Furthermore, Jürgens and Takano et al. [23, 24] discovered that hypoxia decreased the expression of CYP2D1, CYP3A1, and CYP2B1 in normal and healthy subject hepatocytes, and this is consistent with the trend of decreased CYP2D1 and CYP2B1 in this experiment.

However, the results of DIA sequencing and Western blot analysis for Abcg2 and CYP3A1 were opposite in this experiment, and certain discrepancies were observed between Slco1a2 protein and mRNA expression in the blood-brain barrier and hCMEC/D_3_ under hypoxic conditions. We hypothesize that these inconsistencies may arise from the following three aspects: First, DIA is an unbiased global proteomic screening approach, and its quantification is based on peptide signal intensity. For proteins with low abundance or post-translational modifications, the quantitative accuracy may be affected to some extent. In contrast, RT-qPCR and Western blot are targeted validation methods with higher sensitivity and specificity, enabling more precise detection of expression changes in specific genes or proteins. Furthermore, changes at the transcriptional level do not necessarily correspond to alterations at the protein level, as intermediate regulatory processes such as translational inhibition, post-transcriptional regulation, and enhanced protein degradation may be involved. Finally, the differences in sample types between rat brain tissue and hCMEC/D_3_ may also contribute to discrepancies between protein and mRNA expression levels. These experimental discrepancies represent a limitation of the present study, yet they also constitute one of its findings, suggesting that the changes in protein and mRNA expression under hypoxic conditions involve a complex and dynamic regulatory process. Further studies are warranted to confirm these observations. Additionally, sequencing studies concluded that Tjp1 is the most significant tight-junction protein in hypoxic environments and that Tjp1 alterations are essential for drug trafficking and barrier integrity. Based on this, we also examined the Tjp1 protein and mRNA expression in rat brain tissues and hCMEC/D_3_. The results demonstrated that the Tjp1 protein and mRNA expression was decreased, implying that more permeability in hypoxic environments of the blood-brain barrier may be related to decreased tight-junction expression.

We carried out DIA, western blot, and RT-qPCR analyses to evaluate changes in the expression of major drug transporters and metabolizing enzymes that are likely to be relevant for drug metabolism under high-altitude hypoxia conditions. Specific drugs substrate were administered to evaluate transporter and enzyme function, thereby enabling further investigation into the effects of high-altitude hypoxia on drug pharmacokinetics. The efflux transporter Abcb1 and the uptake transporter Slco1a2 are key targets in blood-brain barrier drug transport research. Inhibition of Abcb1 or structural modification of drugs to mimic Abcb1 substrates may enhance drug delivery into the central nervous system by evading Abcb1-mediated efflux. Meanwhile, Slco1a2 facilitates the transmembrane transport of numerous drugs via a sodium-independent mechanism. Additionally, the drug-metabolizing enzyme CYP2B1, which is highly expressed in brain tissue, plays an important role in reducing intracerebral drug accumulation [25–27]. Consequently, our research was focused on the drugs substrate of Abcb1, Slco1a2, and CYP2B1-propranolol hydrochloride, zolmitriptan, and verapamil hydrochloride. We observed that the pharmacokinetic parameters of these drugs were altered under both acute and chronic hypoxic conditions. These changes suggest a reduction in the activities of Abcb1, Slco1a2, and CYP2B1 in the high altitude hypoxia. Furthermore, in line with the decreased metabolic clearance observed under hypoxic conditions, *t*_1/2_ for all three drug substrates increased in hypoxic rats. Chai et al. reported that hypoxic conditions modified the pharmacokinetic parameters of medications used to treat central nervous system -related diseases and downregulated Abcb1 expression and activity at the at the blood-brain barrier [28]. Burgess et al. [29] demonstrated that the rs70950385 mutation enhances mRNA stability through the disruption of the endogenous miR-1275 binding site in the CYP2B1 3‘UTR, providing a mechanistic explanation for the ultra-rapid CYP2B1 metabolic phenotype observed in some normal metabolizers. Nevertheless, variations in CYP2B1 activity across distinct brain regions remain poorly characterized. Our previous findings that hypoxia significantly increased Abcb1 and Abcg2 transporter activities in Caco-2 cells contrast with the results obtained from the present in vivo study [30]. However, these results consistent with the observed upregulated of Abcb1 activity in the uptake transporter assay using hCMEC/D_3_. These discrepancies may stem from inherent differences between cellular-level and in vivo transporter activities of rats, or from variations across different cellular models. Alternatively, they could be attributed to substantial inter-individual variability in the pharmacokinetics of the experimental animals. Further verification through repeated experiments is warranted to clarify these findings. The current studies show that activities of transporters and metabolizing enzymes were altered in rats in vivo and in hCMEC/D_3_, demonstrating that there is a degree of alteration in drug uptake distribution, metabolism, and transport mediated by these transporters and metabolizing enzymes.

Pharmacokinetic analysis in this study demonstrated that high-altitude hypoxia enhanced the blood-brain barrier penetration of propranolol hydrochloride, while markedly reducing the blood-brain barrier transport of zolmitriptan. Meanwhile, in the hypoxic-treated hCMEC/D_3_ monolayer cell model, both the efflux function of Abcb1 and the uptake capacity of Slco1a2 were significantly upregulated. These in vitro functional alterations collectively explained the divergent transport characteristics of the two drugs under hypoxic conditions, namely the increased brain delivery of propranolol hydrochloride and the restricted blood-brain barrier permeability of zolmitriptan. Superficially, the increased blood-brain barrier transport of propranolol under hypoxia appears contradictory to the enhanced Abcb1 efflux function; however, this phenomenon precisely reveals the in vivo-in vitro discrepancies and the complexity of transporter network regulation. Firstly, the transporter “function” measured in cellular experiments refers to the directional transport capacity of a single transporter under standardized substrate conditions. In contrast, in vivo pharmacokinetic parameters reflect the comprehensive outcome of the interactions among multiple transporters, passive diffusion, drug metabolism, plasma protein binding, cerebral blood flow, drug physicochemical properties and other confounding factors. For propranolol, despite hypoxia-mediated upregulation of Abcb1 efflux activity, the concurrent suppression of uptake transporter expression in cerebral microvascular endothelial cells ultimately leads to elevated drug accumulation in brain tissue. Conversely, the reduced brain distribution of zolmitriptan seems inconsistent with the expected outcome of enhanced Slco1a2 function. A plausible explanation is that the elevated efflux activity of Abcb1 exerts a stronger net efflux effect on zolmitriptan than the enhanced uptake mediated by Slco1a2. Additionally, alterations in plasma protein binding or free drug concentration may further disrupt the concentration gradient for blood-brain barrier penetration. Accordingly, the inconsistencies among transporter expression levels, functional measurements in cell monolayers, and in vivo pharmacokinetic results do not invalidate the proposed mechanistic framework. Instead, these findings indicate that hypoxia induces functional remodeling of drug transporters at the blood-brain barrier, providing critical insights for further exploration of the molecular mechanisms underlying substrate-specific differential drug transport under hypoxic conditions. However, further investigation is required to determine whether populations residing on the Tibetan Plateau experience the same alterations in their central nervous system drug transporters and metabolizing enzymes as rats.

In addition to the alterations in pharmacokinetic parameters, we also analyzed the underlying cause of the increased brain drug concentration-specifically, whether it is primarily due to tight junction leakage, reduced active efflux, or reduced metabolic enzyme activity-by integrating the aforementioned findings of increased Evans blue dye extravasation and changes in the brain/blood partition coefficient. we comparatively analyzed alterations in the Kp among the three tested agents. The results demonstrated that the magnitude of Kp elevation was markedly greater for propranolol hydrochloride, a typical Abcb1 substrate, than for zolmitriptan, a substrate of Slco1a2. Considering that blood-brain barrier structural damage generally induces non-selective increases in passive permeability, exclusive tight junction disruption would theoretically cause a comparable rise in Kp across all experimental drugs. Accordingly, the distinct inter-agent discrepancy in Kp variation strongly suggests that suppressed Abcb1-dependent active efflux is the primary mechanism driving the intracellular accumulation of its substrate in brain tissue. In parallel, evident Evans blue extravasation validated the destruction of blood-brain barrier structural integrity, which provides a pathological foundation for enhanced passive transendothelial penetration of small-molecule drugs and acts as a synergistic factor facilitating cerebral drug infiltration. Moreover, the downregulated expression of cerebral CYP2B1 diminished local metabolic clearance within the brain, further prolonging the elimination half-life of the tested compounds. Collectively, the hypoxia-induced elevation in cerebral drug exposure arises from the combined effects of diminished active efflux, compromised barrier integrity, and attenuated in-situ cerebral metabolism. Of these regulatory mechanisms, the functional suppression of efflux transporters occupies a dominant position in modulating brain drug distribution. Thus, focusing on functional alterations of drug transporters is crucial for understanding how high-altitude hypoxia affects the distribution of central nervous system drugs.

Central nervous system drugs to have an effect, they need to cross the blood-brain barrier and enter the brain. This indicates that the ratio of blood to brain drug concentration must remain relatively stable in order for the drug to be used correctly. If this ratio is altered, administering drugs at their current dosages could pose unintended risks to patients and compromise the effectiveness of drug therapy [31, 32]. The purpose of the current study was conducted to evaluate the effects of plateau hypoxia on the cerebral blood distribution of propranolol hydrochloride, zolmitriptan and verapamil hydrochloride by oral administration. Results demonstrated that high-altitude hypoxia markedly altered the brain distribution and blood-brain barrier permeability of the three tested drugs in rats. To a certain extent, these alterations were associated with the abnormal expression of the efflux transporter Abcb1 and the uptake transporter Slco1a2, as well as the downregulation of the metabolic enzyme CYP2B1. In addition, structural damage and functional impairment of the blood-brain barrier, together with potential crosstalk between transporters and metabolic enzymes, jointly contributed to the observed phenotypic changes. Notably, high-altitude hypoxia also triggers extensive systemic adaptive responses, including alterations in hepatic metabolism, renal clearance, systemic blood redistribution and plasma protein binding. Accordingly, the overall pharmacokinetic changes of the tested drugs result from the combined effects of local blood-brain barrier regulation and systemic peripheral physiological adaptation, rather than being solely attributed to the modulation of cerebral transporters and metabolic enzymes. In future studies, we will adopt in situ brain perfusion, isolated organ models and transporter-specific intervention approaches to distinguish central blood-brain barrier specific effects from peripheral metabolic and clearance changes. This will help to clarify the respective regulatory mechanisms of central and peripheral pathways, and systematically elucidate their influences on the in vivo kinetic profiles of drugs under hypoxic conditions. Given that targeted mechanistic interventional experiments, such as gene knockdown and pharmacological inhibition, were not performed in the present study, the definite causal relationships between phenotypic changes and these regulatory factors cannot be fully established. The specific molecular regulatory mechanisms remain to be clarified in further investigations. Nevertheless, the observed differences in brain-blood drug distribution in this study indicate that drug efficacy may be altered under hypoxic conditions, and the specific mechanisms underlying such changes require further in-depth exploration.

In addition to the inherent limitations of the experimental model, all in vivo experiments in this study were conducted under natural hypoxic conditions at an altitude of 4300 m. Accordingly, the quantitative results are inevitably condition-dependent and difficult to be fully reproduced by other researchers. We have comprehensively reviewed and analyzed relevant domestic and international studies as follows. Dopp et al. adopted a hypobaric chamber to simulate intermittent hypoxia and confirmed that hypoxia upregulated the expression of Abcb1 in the myocardium and liver tissues of SD rats [33]. The research team led by Fradette from the University of Montreal exposed rabbits to acute hypoxia with 8% oxygen concentration and found a significant elevation in CYP3A expression [34]. Zhou et al. reported that long-term exposure of SD rats to high-altitude environments at 2800 m and 3400 m markedly decreased the mRNA and protein levels of hepatic CYP3A1 [35]. Duan et al. further demonstrated that the expression and activity of Abcg2 and OATP2B1 in the liver and small intestines were significantly altered under natural hypoxic conditions at 2800 m and 4300 m [13, 30]. Moreover, Li et al. verified that high-altitude hypoxia at 2800 m and 4300 m notably suppressed the expression and activity of hepatic CYP3A1, while distinctly increasing the levels of CYP2D1 and CYP2B1 in rats [12, 36]. Currently, studies on drug transporters and metabolizing enzymes in brain tissue under high-altitude conditions remain relatively limited. Nevertheless, the above-mentioned investigations conducted in different countries, at various altitudes, using distinct hypoxia models and experimental species consistently demonstrate that hypoxic environments can significantly regulate the expression and function of drug transporters and metabolizing enzymes, indicating that such adaptive regulatory patterns exhibit cross-regional conservation. Although differences in the duration of hypoxia exposure, intervention intensity, and animal strains may lead to variations in the magnitude of the effects, the core trends are generally consistent. Therefore, the principal conclusions of this study are not confined to a single region or a domestic experimental system; rather, they fundamentally reflect the common physiological characteristics of drug metabolism and transport regulation across multiple tissues under hypoxic stress. At the same time, we fully recognize that confounding factors such as animal sources, housing conditions, dietary protocols, and hypoxia intervention modalities across different regions may cause quantitative discrepancies and limited reproducibility. Consequently, further validation using multi-altitude field studies, artificial hypoxia models, and multiple species is required to refine and confirm the broad applicability of our findings.

The present study has several inherent limitations. First, SD rats and the hCMEC/D_3_ line cannot fully recapitulate the complex physiological functions of the human blood-brain barrier. Significant interspecies differences between rodents and humans exist in the expression profiles and substrate specificity of drug transporters. Second, this investigation focuses on acute and chronic high-altitude hypoxic exposure, whereas long-term highland residents develop stable physiological adaptation via multi-system compensatory regulation. Such distinct adaptive mechanisms lead to divergent molecular regulatory patterns, restricting the direct extrapolation of our findings to chronically adapted high-altitude populations. In addition, hCMEC/D_3_ exhibit obvious discrepancies in transporter activity, tight junction integrity and hypoxic responsiveness compared with primary human brain microvascular endothelial cells. Finally, targeted validations, including gene knockdown, pharmacological intervention and specific transporter blockade, were not performed in this study, resulting in a lack of direct causal evidence for the underlying molecular mechanisms. Collectively, the recommendations for clinical medication adjustment in high-altitude regions proposed in this study are merely preliminary experimental inferences. Further investigations utilizing physiologically relevant humanized preclinical models, combined with in-depth mechanistic validation, are warranted to confirm our findings and evaluate their clinical translational potential.

## Conclusions

In summary, this study explores the structural and functional alterations of the blood-brain barrier under high-altitude hypoxia, as well as their relationship with drug transport mechanisms. Furthermore, it proposes a hypothesis framework termed “high-altitude hypoxia - blood-brain barrier - drug transporters - drug-metabolizing enzymes - drug transport”. This hypothesis provides novel insights into the research of drug metabolic mechanisms under high-altitude hypoxia, and also offers preliminary experimental references for the prevention and treatment of central nervous system diseases as well as individualized clinical treatment in high-altitude regions.

## Electronic supplementary material

Below is the link to the electronic supplementary material.

Supplementary material 1

Supplementary material 2

## Data Availability

No datasets were generated or analysed during the current study.

## Abbreviations

DIA: Data-independent acquisition
DDA: Data-dependent acquisition
*t*₁/₂: Half-life
CL: Total clearance
*C*_brain_/*C*_plasma_: Brain-blood drug partition ratio
Kp_(AUCbrain/AUCplasma)_: Blood-brain barrier permeability coefficient
ABC families: ATP-Binding cassette transporter superfamily
SLC families: Solute carrier family transporters
GSEA analysis: Gene set enrichment analysis
UPLC-MS: Ultra performance liquid chromatography-mass spectrometry
C_max_: Maximum plasma concentration
T_max_: Peak time
AUC: Area under the concentration time curve
V_d_: Apparent volume of distribution
MRT: Mean residence time
Abcb1: ATP Binding Cassette Subfamily B Member 1
Slco1a2: Solute Carrier Organic Anion Transporter Family Member 1A2
Abcg2: ATP Binding Cassette Subfamily G Member 2
