# Supplementary material for: Functional impact of high-altitude hypoxia on central nervous system drug transport: integrated analysis of blood-brain barrier permeability, drug transporter/enzyme expression, and pharmacokinetics
Source: Fluids Barriers CNS. 2026 Jun 11;23:100. doi: 10.1186/s12987-026-00829-y (PMC13501568; doi:10.1186/s12987-026-00829-y)
Supplement: Supplementary file 1 [file 12987_2026_829_MOESM1_ESM.docx]

**Supporting Information**

**Table S1** Primer sequences corresponding to genes detected by RT-qPCR in blood-brain barrier

| Gene | Primer | Primer sequence (5’-3’) |
| --- | --- | --- |
| Abcb1 | forward | 5'-CTCGCTGCTATCATCCACGGAAC-3' |
|  | reverse | 5'-CGCTGACGGTCTGTGTACTGTTG-3' |
| Abcg2 | forward | 5'-TTAGGACTGAAGAGGACGGTGGAG-3' |
|  | reverse | 5'-TTGCTACAGACACTACGCTTTGGC-3' |
| Slco1a2 | forward | 5' - AAGACCAACGCAGGATCCAT- 3' |
|  | reverse | 5' - GAGTTTCACCCATTCCACGTACA- 3' |
| CYP2B1 | forward | 5' - TTTGGTGGAGGAACTGCGGAAATC- 3' |
|  | reverse | 5' - AGGAACTGGCGGTCTGTGTAGTC- 3' |
| CYP2D1 | forward | 5' - TGGAGCAGTGGGTGACAGA- 3' |
|  | reverse | 5' - AAGTCCAGGAGCCTGATGAA- 3' |
| CYP3A1 | forward | 5' - CGTTCACCAGTGGAAGACTCAAGG - 3' |
|  | reverse | 5' - TTCTTTCACAGGGACAGGTTTGCC - 3' |
| Tjp1 | forward | 5′-TAAACCTCCAAGTGCTTCCCT-3′ |
|  | reverse | 5′-CTTCAGGTGGCTTGACTTGAG-3′ |
| β-actin | forward | 5'-TCACCAACTGGGACGATATG-3' |
|  | reverse | 5'-GTTGGCCTTAGGGTTCAGAG-3' |

**Table S2** Primer sequences corresponding to genes detected by RT-qPCR in hCMEC/D_3_

| Gene | Primer | Primer sequence (5’-3’) |
| --- | --- | --- |
| Abcb1 | forward | 5′-TGCTCAAGTTAAAGGGGCTA-3' |
|  | reverse | 5′-CAGTGTTAGTTGCCAACCAT-3' |
| Abcg2 | forward | 5′-TAGCTGCAAGGAAAGATCCAA-3' |
|  | reverse | 5′-TCATCTTGTACCACGTAACCTG-3' |
| Slco1a2 | forward | 5′-TCATAACTCCCACTGACACT-3' |
|  | reverse | 5′-GTTAACTCCTGCACAAATCAGA-3' |
| CYP2B6 | forward | 5′-TTTGCTGGCACTGAGACCACC-3' |
|  | reverse | 5′-ATTTTGGCTCGGTCATGAAGCTC-3' |
| CYP2D6 | forward | 5′-TTTGTGCCGCCTTCGCCAACCA-3' |
|  | reverse | 5′-CGATCACGTTGCTCACGGCTTT-3' |
| CYP3A4 | forward | 5′-ATTGATGCAGTTTTACCCAA-3' |
|  | reverse | 5′-TAATCTGAGCGTTTCATTCACC-3' |
| Tjp1 | forward | 5′-TAAACCTCCAAGTGCTTCCCT-3′ |
|  | reverse | 5′-CTTCAGGTGGCTTGACTTGAG-3′ |
| β-actin | forward | 5′-TCCTCCTGAGCGCAAGTACTCC-3′ |
|  | reverse | 5′-CATACTCCTGCTTGCTGATCCAC-3′ |

**Table S3** Drug transporters in the blood-brain barrier under high-altitude hypoxia

| Protein_id | DIA_Qvalue | Symbol | LC | AH | CH |
| --- | --- | --- | --- | --- | --- |
| ENSRNOG00000000455 | 0.0000 | Tap2 | 14.5842 | 28.9221 | 28.9256 |
| ENSRNOG00000000465 | 0.0000 | Slc39a7 | 24.6729 | 11.5384 | 11.6536 |
| ENSRNOG00000000799 | 0.0000 | Abcf1 | 242.0176 | 281.6769 | 231.4804 |
| ENSRNOG00000000878 | 0.0000 | Slc44a4 | 137.0186 | 68.3604 | 0.0000 |
| ENSRNOG00000000879 | 0.0000 | Slc9a6 | 275.9314 | 252.8704 | 284.9029 |
| ENSRNOG00000000924 | 0.0000 | Slc7a1 | 274.0627 | 342.7922 | 321.3880 |
| ENSRNOG00000001082 | 0.0000 | Abcb9 | 116.2428 | 78.7060 | 82.6285 |
| ENSRNOG00000001158 | 0.0039 | Abcg1 | 83.2379 | 100.9883 | 67.2713 |
| ENSRNOG00000001298 | 0.0004 | Slc5a4b | 16.5409 | 9.8318 | 8.5117 |
| ENSRNOG00000001492 | 0.0000 | Slc8a2 | 1713.5286 | 1979.7067 | 2912.3385 |
| ENSRNOG00000001710 | 0.0000 | Abcf3 | 250.9200 | 288.9804 | 253.9212 |
| ENSRNOG00000001874 | 0.0000 | Slc7a4 | 19.9584 | 39.3817 | 23.7129 |
| ENSRNOG00000002246 | 0.0000 | Slc30a9 | 596.4463 | 506.5855 | 563.6776 |
| ENSRNOG00000002305 | 0.0000 | Slc15a2 | 455.3650 | 491.3038 | 673.3260 |
| ENSRNOG00000002790 | 0.0000 | Abcb7 | 290.1181 | 319.5429 | 300.8958 |
| ENSRNOG00000002948 | 0.0000 | Abcc3 | 906.0146 | 1148.9564 | 1375.9886 |
| ENSRNOG00000002997 | 0.0000 | Slc9a3r2 | 507.9814 | 484.9952 | 439.5255 |
| ENSRNOG00000003134 | 0.0000 | Slc4a4 | 2680.1235 | 2206.7709 | 1719.6345 |
| ENSRNOG00000003232 | 0.0000 | Slc9a3r1 | 2918.0432 | 3384.3326 | 3590.5470 |
| ENSRNOG00000003476 | 0.0000 | Slc6a4 | 398.1768 | 286.9616 | 316.5701 |
| ENSRNOG00000003815 | 0.0000 | Slc25a11 | 9255.6452 | 8965.5669 | 11046.0111 |
| ENSRNOG00000003835 | 0.0000 | Slc43a2 | 173.7823 | 152.2334 | 150.2217 |
| ENSRNOG00000003918 | 0.0000 | Slc25a19 | 224.1757 | 224.6330 | 221.5408 |
| ENSRNOG00000004150 | 0.0000 | Slc9a7 | 323.2645 | 232.4830 | 288.0580 |
| ENSRNOG00000004351 | 0.0000 | Slc25a29 | 120.6432 | 126.6516 | 112.6679 |
| ENSRNOG00000004378 | 0.0000 | Abca5 | 125.9737 | 106.7677 | 98.8489 |
| ENSRNOG00000004510 | 0.0000 | Slc35b1 | 107.0233 | 140.2700 | 123.9016 |
| ENSRNOG00000004668 | 0.0000 | Slc25a35 | 309.1126 | 496.4530 | 301.4147 |
| ENSRNOG00000004749 | 0.0000 | Slc30a1 | 331.3435 | 415.4614 | 387.7994 |
| ENSRNOG00000004901 | 0.0000 | Slc4a1ap | 127.9541 | 139.3148 | 120.6996 |
| ENSRNOG00000005196 | 0.0000 | Slc12a6 | 3663.3183 | 3793.8857 | 4365.6873 |
| ENSRNOG00000005248 | 0.0000 | Slc1a4 | 2324.5722 | 2160.0085 | 2345.9654 |
| ENSRNOG00000005291 | 0.0000 | Slc38a1 | 65.2164 | 68.6180 | 53.4912 |
| ENSRNOG00000005307 | 0.0000 | Slc4a10 | 2408.2277 | 3006.0229 | 3162.8871 |
| ENSRNOG00000005479 | 0.0000 | Slc1a2 | 14020.5267 | 17337.6309 | 20824.0260 |
| ENSRNOG00000005697 | 0.0000 | Slc6a11 | 13449.8050 | 13077.6216 | 7567.2424 |
| ENSRNOG00000005957 | 0.0000 | Slc4a7 | 82.4483 | 100.6226 | 88.5851 |
| ENSRNOG00000006204 | 0.0000 | Slc30a3 | 1930.4146 | 2653.1749 | 3387.5347 |
| ENSRNOG00000006305 | 0.0000 | Slc38a2 | 44.4236 | 31.2847 | 24.7478 |
| ENSRNOG00000006527 | 0.0000 | Slc6a1 | 1725.0684 | 1731.4986 | 1730.2480 |
| ENSRNOG00000006871 | 0.0000 | Slc25a14 | 128.1264 | 147.2356 | 150.5566 |
| ENSRNOG00000007041 | 0.0000 | Abcg2 | 121.4084 | 104.4724 | 75.8589 |
| ENSRNOG00000007284 | 0.0000 | Slc2a1 | 4209.4178 | 4479.6958 | 4753.7124 |
| ENSRNOG00000007509 | 0.0000 | Slc1a6 | 11415.2717 | 12371.3857 | 9553.0081 |
| ENSRNOG00000007839 | 0.0000 | Slc16a7 | 269.7413 | 473.3529 | 432.4712 |
| ENSRNOG00000007982 | 0.0000 | Slc9a1 | 668.1803 | 556.0717 | 598.0989 |
| ENSRNOG00000008012 | 0.0000 | Abcb1 | 510.0709 | 504.9099 | 635.5742 |
| ENSRNOG00000008169 | 0.0000 | Slc24a2 | 775.5593 | 781.5501 | 983.2604 |
| ENSRNOG00000008243 | 0.0000 | Slc43a1 | 88.5487 | 87.8433 | 95.6861 |
| ENSRNOG00000008376 | 0.0000 | Slc2a3 | 2101.4956 | 2031.4196 | 2801.7132 |
| ENSRNOG00000008479 | 0.0000 | Slc8a1 | 2181.1573 | 2294.4733 | 2147.2031 |
| ENSRNOG00000008557 | 0.0000 | Abcb8 | 492.1710 | 469.5747 | 516.6784 |
| ENSRNOG00000008851 | 0.0000 | Slc35b4 | 144.5032 | 121.9944 | 138.8679 |
| ENSRNOG00000008890 | 0.0000 | Slc18a2 | 50.3037 | 53.5216 | 41.3783 |
| ENSRNOG00000009019 | 0.0000 | Slc6a6 | 114.3345 | 161.9430 | 127.6396 |
| ENSRNOG00000009459 | 0.0000 | Slc35f6 | 69.2066 | 62.3637 | 64.3925 |
| ENSRNOG00000009740 | 0.0000 | Slco1c1 | 52.5228 | 51.4885 | 63.9921 |
| ENSRNOG00000009957 | 0.0000 | Slc25a13 | 9688.3913 | 9115.8928 | 6290.2471 |
| ENSRNOG00000010023 | 0.0000 | Slc33a1 | 247.9659 | 202.1311 | 217.1170 |
| ENSRNOG00000010064 | 0.0000 | Abcc4 | 113.6908 | 179.1439 | 123.5894 |
| ENSRNOG00000010128 | 0.0000 | Slc27a2 | 79.9386 | 120.7787 | 48.4885 |
| ENSRNOG00000010210 | 0.0000 | Slc7a11 | 552.8547 | 636.7585 | 765.5695 |
| ENSRNOG00000010592 | 0.0000 | Slc25a27 | 297.8429 | 330.7314 | 409.1364 |
| ENSRNOG00000010597 | 0.0000 | Slc5a7 | 288.6524 | 345.1240 | 292.9278 |
| ENSRNOG00000010609 | 0.0000 | Abcf2 | 377.1257 | 316.1824 | 329.0333 |
| ENSRNOG00000010830 | 0.0000 | Slc25a4 | 75884.2109 | 74001.1524 | 76522.7396 |
| ENSRNOG00000010938 | 0.0000 | Slc7a10 | 512.8933 | 281.1625 | 312.5920 |
| ENSRNOG00000011016 | 0.0000 | Slc7a2 | 87.2022 | 89.1913 | 87.0139 |
| ENSRNOG00000011184 | 0.0000 | Slc13a4 | 67.2038 | 61.5262 | 50.0096 |
| ENSRNOG00000011361 | 0.0000 | Slc37a4 | 145.2322 | 104.2765 | 110.4104 |
| ENSRNOG00000011455 | 0.0014 | Slc36a4 | 35.6318 | 26.3024 | 24.9508 |
| ENSRNOG00000011677 | 0.0000 | Slc39a10 | 517.9071 | 564.0814 | 672.1067 |
| ENSRNOG00000011929 | 0.0000 | Abcd3 | 552.2535 | 477.8468 | 399.1432 |
| ENSRNOG00000012287 | 0.0000 | Slc35e1 | 34.0766 | 36.2807 | 30.4273 |
| ENSRNOG00000012356 | 0.0000 | Slc36a1 | 57.1270 | 88.2705 | 49.4828 |
| ENSRNOG00000012831 | 0.0000 | Slc25a26 | 72.2712 | 103.7311 | 80.5362 |
| ENSRNOG00000012876 | 0.0000 | Slc6a13 | 109.0642 | 95.9529 | 63.3135 |
| ENSRNOG00000012939 | 0.0000 | Abca7 | 43.6315 | 53.7216 | 54.9975 |
| ENSRNOG00000013407 | 0.0000 | Slc5a11 | 50.1498 | 20.5565 | 21.0987 |
| ENSRNOG00000013802 | 0.0000 | Slc25a36 | 218.5505 | 201.6344 | 185.9260 |
| ENSRNOG00000014268 | 0.0000 | Abca2 | 344.5696 | 228.6934 | 229.9692 |
| ENSRNOG00000014311 | 0.0000 | Slc7a8 | 192.5549 | 243.2995 | 280.9278 |
| ENSRNOG00000014338 | 0.0000 | Slc25a25 | 787.9210 | 438.0470 | 464.1349 |
| ENSRNOG00000014347 | 0.0000 | Slc4a2 | 232.1999 | 226.8816 | 158.9122 |
| ENSRNOG00000014369 | 0.0000 | Slc27a4 | 618.9767 | 543.2733 | 579.2213 |
| ENSRNOG00000014475 | 0.0000 | Slc31a1 | 128.6923 | 95.2052 | 105.1758 |
| ENSRNOG00000014816 | 0.0000 | Slc1a1 | 151.9912 | 160.0467 | 185.2067 |
| ENSRNOG00000014870 | 0.0000 | Slc13a5 | 100.5452 | 115.2386 | 117.1469 |
| ENSRNOG00000015370 | 0.0000 | Slc35g2 | 135.1065 | 99.1476 | 101.1914 |
| ENSRNOG00000015393 | 0.0000 | Slc32a1 | 3547.2136 | 4091.9625 | 3166.5448 |
| ENSRNOG00000015421 | 0.0000 | Slc27a3 | 147.7639 | 146.2131 | 152.0718 |
| ENSRNOG00000015538 | 0.0000 | Abcd2 | 244.9151 | 297.2798 | 201.2087 |
| ENSRNOG00000015741 | 0.0000 | Slc2a13 | 284.6820 | 254.9438 | 300.1221 |
| ENSRNOG00000015948 | 0.0000 | Slc1a5 | 16.5807 | 31.9749 | 29.1775 |
| ENSRNOG00000015971 | 0.0000 | Slc12a2 | 2226.3080 | 1633.8974 | 1342.2274 |
| ENSRNOG00000016147 | 0.0000 | Slc17a6 | 721.4941 | 527.5208 | 550.3106 |
| ENSRNOG00000016163 | 0.0000 | Slc1a3 | 7310.8197 | 11079.0562 | 8226.3107 |
| ENSRNOG00000016414 | 0.0000 | Slc22a17 | 447.0291 | 351.0749 | 398.2567 |
| ENSRNOG00000016753 | 0.0000 | Slc14a1 | 540.1586 | 387.9521 | 386.8125 |
| ENSRNOG00000016827 | 0.0000 | Slc38a3 | 877.9468 | 948.7484 | 841.6646 |
| ENSRNOG00000017091 | 0.0000 | Slc25a46 | 366.5469 | 367.1018 | 367.1657 |
| ENSRNOG00000017210 | 0.0000 | Slc22a23 | 543.9195 | 567.3842 | 565.0936 |
| ENSRNOG00000017302 | 0.0000 | Slc6a3 | 508.5269 | 800.8599 | 432.0517 |
| ENSRNOG00000017693 | 0.0000 | Slc2a5 | 42.7340 | 65.1966 | 50.0691 |
| ENSRNOG00000017993 | 0.0000 | Abcb10 | 187.0237 | 168.8766 | 176.8272 |
| ENSRNOG00000018086 | 0.0013 | Slc22a8 | 39.6336 | 45.2559 | 40.1701 |
| ENSRNOG00000018111 | 0.0000 | Slc12a5 | 5345.0482 | 5293.3159 | 6456.0360 |
| ENSRNOG00000018126 | 0.0000 | Abca1 | 110.1735 | 136.1655 | 106.5569 |
| ENSRNOG00000018170 | 0.0000 | Slc27a1 | 646.0887 | 609.8766 | 590.2208 |
| ENSRNOG00000018345 | 0.0000 | Abce1 | 883.0037 | 930.1313 | 815.2483 |
| ENSRNOG00000018487 | 0.0000 | Slc3a2 | 5077.2497 | 4576.3293 | 5128.5721 |
| ENSRNOG00000018567 | 0.0000 | Slc20a1 | 342.2254 | 338.9978 | 328.5708 |
| ENSRNOG00000018644 | 0.0000 | Slc6a7 | 887.2343 | 855.0821 | 1110.0883 |
| ENSRNOG00000018697 | 0.0000 | Abcb6 | 184.4105 | 167.2696 | 146.6123 |
| ENSRNOG00000018746 | 0.0000 | Slc30a5 | 113.3786 | 97.6802 | 101.5668 |
| ENSRNOG00000018785 | 0.0000 | Slc16a13 | 59.8220 | 54.0285 | 61.0354 |
| ENSRNOG00000018824 | 0.0000 | Slc7a5 | 486.4513 | 444.1063 | 444.8160 |
| ENSRNOG00000018863 | 0.0000 | Abcc10 | 8.0048 | 14.7787 | 6.1802 |
| ENSRNOG00000018920 | 0.0000 | Slc25a17 | 3062.4670 | 2337.2783 | 2171.9587 |
| ENSRNOG00000019118 | 0.0000 | Slc13a3 | 144.3329 | 79.6042 | 95.1985 |
| ENSRNOG00000019484 | 0.0000 | Slc6a9 | 2181.0744 | 1010.4038 | 939.3273 |
| ENSRNOG00000019490 | 0.0000 | Slc20a2 | 189.0570 | 158.4575 | 160.4659 |
| ENSRNOG00000019651 | 0.0000 | Slc12a4 | 95.8338 | 100.8125 | 101.0161 |
| ENSRNOG00000019752 | 0.0000 | Slc29a1 | 182.4166 | 199.5752 | 188.7686 |
| ENSRNOG00000019996 | 0.0000 | Slc16a1 | 1317.6102 | 991.4618 | 1013.9410 |
| ENSRNOG00000020025 | 0.0000 | Slc29a2 | 24.1386 | 32.0986 | 23.4973 |
| ENSRNOG00000020138 | 0.0000 | Slc4a3 | 151.9205 | 161.1582 | 167.8226 |
| ENSRNOG00000020288 | 0.0000 | Slc25a20 | 847.5808 | 733.2687 | 672.2078 |
| ENSRNOG00000020345 | 0.0000 | Slc25a42 | 287.0640 | 302.0512 | 334.4460 |
| ENSRNOG00000020450 | 0.0000 | Slc26a6 | 19.0852 | 26.4353 | 17.1377 |
| ENSRNOG00000020470 | 0.0000 | Slc25a24 | 325.5937 | 370.2775 | 351.5214 |
| ENSRNOG00000020650 | 0.0000 | Slc17a7 | 7675.7253 | 9173.2095 | 13473.8233 |
| ENSRNOG00000020951 | 0.0000 | Slc4a1 | 2584.4375 | 2295.0868 | 2624.7117 |
| ENSRNOG00000021130 | 0.0000 | Abcc8 | 76.8373 | 94.7805 | 98.3882 |
| ENSRNOG00000021262 | 0.0000 | Slc23a2 | 115.6389 | 151.0793 | 145.8533 |
| ENSRNOG00000022239 | 0.0000 | Trarg1 | 32.6567 | 169.8832 | 0.0000 |
| ENSRNOG00000022305 | 0.0000 | Abcc1 | 162.7236 | 184.1724 | 165.1315 |
| ENSRNOG00000022837 | 0.0000 | Slc25a40 | 33.3293 | 44.2368 | 45.8006 |
| ENSRNOG00000022922 | 0.0000 | Slc25a12 | 9543.5391 | 8512.8899 | 10463.9958 |
| ENSRNOG00000024801 | 0.0000 | Slc35a2 | 32.1997 | 19.6537 | 16.9438 |
| ENSRNOG00000025269 | 0.0000 | Slc25a44 | 95.3201 | 111.9111 | 106.3374 |
| ENSRNOG00000025639 | 0.0000 | Slc39a12 | 12.1718 | 24.5410 | 0.0000 |
| ENSRNOG00000026091 | 0.0000 | Slc10a4 | 15.8733 | 26.7370 | 19.6181 |
| ENSRNOG00000027468 | 0.0000 | Slc6a15 | 32.3695 | 32.2870 | 34.1678 |
| ENSRNOG00000027622 | 0.0003 | Slc35e3 | 2844.2000 | 1855.5663 | 2497.6961 |
| ENSRNOG00000028097 | 0.0000 | Slc7a14 | 1051.9341 | 694.2177 | 988.3847 |
| ENSRNOG00000028703 | 0.0000 | Slc39a6 | 148.5906 | 167.2289 | 135.0871 |
| ENSRNOG00000028879 | 0.0000 | Slc4a8 | 207.5755 | 185.9111 | 198.0759 |
| ENSRNOG00000029178 | 0.0000 | Abcc5 | 39.3312 | 48.3214 | 36.8954 |
| ENSRNOG00000029871 | 0.0000 | Slc8a3 | 120.8182 | 113.7444 | 97.4478 |
| ENSRNOG00000031662 | 0.0000 | Slc6a5 | 623.3288 | 50.4542 | 131.2122 |
| ENSRNOG00000031824 | 0.0000 | Slc44a2 | 427.3626 | 460.2325 | 438.6115 |
| ENSRNOG00000036678 | 0.0000 | Aspscr1 | 592.2739 | 556.6623 | 548.1036 |
| ENSRNOG00000036693 | 0.0000 | Slc25a10 | 244.4784 | 241.3271 | 241.9231 |
| ENSRNOG00000038001 | 0.0000 | Slc25a1 | 2009.9029 | 1420.7387 | 1319.2316 |
| ENSRNOG00000038398 | 0.0001 | Slc25a31 | 699.4402 | 787.3923 | 809.0814 |
| ENSRNOG00000039980 | 0.0000 | Slc25a5 | 57605.3294 | 64197.4453 | 63564.0469 |
| ENSRNOG00000042320 | 0.0000 | Slc41a1 | 11.2396 | 17.8087 | 9.9574 |
| ENSRNOG00000042387 | 0.0000 | Slc25a6 | 369.8919 | 87.2711 | 90.4879 |
| ENSRNOG00000042731 | 0.0000 | Slc25a18 | 10454.8460 | 9386.5715 | 9077.5215 |
| ENSRNOG00000045821 | 0.0000 | Slc41a3 | 249.1489 | 236.7824 | 181.3825 |
| ENSRNOG00000047493 | 0.0000 | Slco1a4 | 103.0231 | 117.6170 | 122.3276 |
| ENSRNOG00000047781 | 0.0000 | Slc25a23 | 590.0539 | 745.8628 | 747.1591 |
| ENSRNOG00000048487 | 0.0000 | Slc12a9 | 240.2954 | 253.5940 | 225.7112 |
| ENSRNOG00000049944 | 0.0000 | Slc25a22 | 4210.2318 | 4277.7507 | 5333.5703 |
| ENSRNOG00000050057 | 0.0000 | Abca3 | 179.5272 | 192.8462 | 190.7746 |
| ENSRNOG00000050090 | 0.0000 | Slc6a17 | 869.2696 | 909.5998 | 1219.7295 |
| ENSRNOG00000050860 | 0.0000 | Abcb11 | 1987.6914 | 2150.5254 | 2707.9661 |
| ENSRNOG00000053196 | 0.0000 | Slc48a1 | 23.3543 | 18.0854 | 15.0986 |
| ENSRNOG00000055089 | 0.0000 | Slc44a1 | 796.4828 | 621.1407 | 623.7886 |
| ENSRNOG00000056524 | 0.0070 | Abcd1 | 10.6822 | 11.6122 | 8.5311 |
| ENSRNOG00000057404 | 0.0003 | Slc47a1 | 35.7685 | 16.0748 | 31.2643 |
| ENSRNOG00000057628 | 0.0000 | Slc5a6 | 31.9931 | 42.4283 | 38.3732 |
| ENSRNOG00000059326 | 0.0000 | Abca9 | 29.0064 | 57.0890 | 28.9303 |
| ENSRNOG00000061768 | 0.0000 | Slc43a3 | 48.4215 | 101.5916 | 58.5449 |
| ENSRNOG00000063289 | 0.0000 | Slc25a5 | 8313.8177 | 6167.2969 | 6741.5605 |
| ENSRNOG00000067988 | 0.0000 | Slc5a3 | 238.0657 | 158.3703 | 183.1384 |
| ENSRNOG00000068001 | 0.0000 | Abcb4 | 79.9557 | 114.8408 | 104.4289 |
| ENSRNOG00000070747 | 0.0000 | Slc25a51 | 108.8902 | 112.0646 | 117.4624 |

Note: protein_id: protein name, DIA_Qvalue: protein identification Q-value, Symbol: protein symbol, LC: Low-altitude control group, AH: High-altitude acute hypoxia group, CH: High-altitude chronic hypoxia group.

**Table S4** Drug-metabolism enzyme in the blood-brain barrier under high-altitude hypoxia

| Protein_id | DIA_Qvalue | Symbol | LC | AH | CH |
| --- | --- | --- | --- | --- | --- |
| ENSRNOG00000004786 | 0.0000 | CYP4F1 | 16.1193 | 36.9025 | 26.8395 |
| ENSRNOG00000004786 | 0.0000 | CYP4F4 | 16.1193 | 36.9025 | 26.8395 |
| ENSRNOG00000007147 | 0.0000 | CYP46A1 | 601.1437 | 789.9497 | 861.3639 |
| ENSRNOG00000007234 | 0.0000 | CYP51 | 416.7180 | 337.7544 | 298.0971 |
| ENSRNOG00000009730 | 0.0000 | CYP7B1 | 137.4232 | 204.9996 | 146.4645 |
| ENSRNOG00000017188 | 0.0000 | CYP27A1 | 5.6178 | 13.8152 | 3.3466 |
| ENSRNOG00000020743 | 0.0000 | CYP2S1 | 60.0694 | 38.8311 | 43.3549 |
| ENSRNOG00000029128 | 0.0000 | CYP2D5 | 257.2359 | 190.2118 | 167.8363 |
| ENSRNOG00000032261 | 0.0000 | CYP2D4(2D6) | 167.6405 | 125.7690 | 116.5836 |
| ENSRNOG00000033164 | 0.0000 | CYP2B3(2B6) | 160.0165 | 105.8351 | 105.1132 |
| ENSRNOG00000042496 | 0.0000 | CYP4F5 | 18.8700 | 16.3500 | 17.1219 |
| ENSRNOG00000042496 | 0.0000 | CYP4F37 | 18.8700 | 16.3500 | 17.1219 |
| ENSRNOG00000043513 | 0.0000 | CYP4X1 | 151.5621 | 205.5183 | 219.0556 |
| ENSRNOG00000055078 | 0.0000 | CYP4B1 | 5.4982 | 21.9957 | 5.1777 |
| ENSRNOG00000066878 | 0.0000 | CYP4A3 | 272.4778 | 547.9329 | 457.7412 |
| ENSRNOG00000067532 | 0.0000 | CYP3A4 | 15.1682 | 16.5921 | 32.2216 |

Note: protein_id: protein name, DIA_Qvalue: protein identification Q-value, Symbol: protein symbol, LC: Low-altitude control group, AH: High-altitude acute hypoxia group, CH: High-altitude chronic hypoxia group.

**Table S5** Prediction of interaction networks of target protein of rats in hypoxia

| Protein_id | Symbol | All degree | Degree | DIA_Qvalue |
| --- | --- | --- | --- | --- |
| ENSRNOG00000000455 | Tap2 | 71 | 71 | 0.0000 |
| ENSRNOG00000000465 | Slc39a7 | 26 | 26 | 0.0000 |
| ENSRNOG00000000799 | Abcf1 | 35 | 35 | 0.0000 |
| ENSRNOG00000000878 | Slc44a4 | 2 | 2 | 0.0000 |
| ENSRNOG00000000879 | Slc9a6 | 39 | 39 | 0.0000 |
| ENSRNOG00000001082 | Abcb9 | 69 | 69 | 0.0000 |
| ENSRNOG00000001158 | Abcg1 | 52 | 52 | 0.0039 |
| ENSRNOG00000001298 | Slc5a4b | 8 | 8 | 0.0004 |
| ENSRNOG00000001492 | Slc8a2 | 10 | 10 | 0.0000 |
| ENSRNOG00000001710 | Abcf3 | 38 | 38 | 0.0000 |
| ENSRNOG00000001874 | Slc7a4 | 63 | 63 | 0.0000 |
| ENSRNOG00000002246 | Slc30a9 | 25 | 25 | 0.0000 |
| ENSRNOG00000002305 | Slc15a2 | 70 | 70 | 0.0000 |
| ENSRNOG00000002948 | Abcc3 | 72 | 72 | 0.0000 |
| ENSRNOG00000002997 | Slc9a3r2 | 33 | 33 | 0.0000 |
| ENSRNOG00000003134 | Slc4a4 | 48 | 48 | 0.0000 |
| ENSRNOG00000003232 | Slc9a3r1 | 41 | 41 | 0.0000 |
| ENSRNOG00000003476 | Slc6a4 | 39 | 39 | 0.0000 |
| ENSRNOG00000003815 | Slc25a11 | 34 | 34 | 0.0000 |
| ENSRNOG00000003835 | Slc43a2 | 20 | 20 | 0.0000 |
| ENSRNOG00000003918 | Slc25a19 | 12 | 12 | 0.0000 |
| ENSRNOG00000004150 | Slc9a7 | 40 | 40 | 0.0000 |
| ENSRNOG00000004351 | Slc25a29 | 6 | 6 | 0.0000 |
| ENSRNOG00000004378 | Abca5 | 47 | 47 | 0.0000 |
| ENSRNOG00000004510 | Slc35b1 | 41 | 41 | 0.0000 |
| ENSRNOG00000004668 | Slc25a35 | 15 | 15 | 0.0000 |
| ENSRNOG00000004749 | Slc30a1 | 50 | 50 | 0.0000 |
| ENSRNOG00000004786 | CYP4F1 | 18 | 18 | 0.0000 |
| ENSRNOG00000032895 | CYP4F4 | 16 | 16 | 0.0000 |
| ENSRNOG00000004901 | Slc4a1ap | 7 | 7 | 0.0000 |
| ENSRNOG00000005196 | Slc12a6 | 54 | 54 | 0.0000 |
| ENSRNOG00000005248 | Slc1a4 | 74 | 74 | 0.0000 |
| ENSRNOG00000005291 | Slc38a1 | 53 | 53 | 0.0000 |
| ENSRNOG00000005307 | Slc4a10 | 24 | 24 | 0.0000 |
| ENSRNOG00000005479 | Slc1a2 | 60 | 60 | 0.0000 |
| ENSRNOG00000005697 | Slc6a11 | 40 | 40 | 0.0000 |
| ENSRNOG00000005957 | Slc4a7 | 35 | 35 | 0.0000 |
| ENSRNOG00000006204 | Slc30a3 | 40 | 40 | 0.0000 |
| ENSRNOG00000006305 | Slc38a2 | 67 | 67 | 0.0000 |
| ENSRNOG00000006527 | Slc6a1 | 37 | 37 | 0.0000 |
| ENSRNOG00000006871 | Slc25a14 | 5 | 5 | 0.0000 |
| ENSRNOG00000007041 | Abcg2 | 86 | 86 | 0.0000 |
| ENSRNOG00000007147 | CYP46A1 | 16 | 16 | 0.0000 |
| ENSRNOG00000007234 | CYP51 | 27 | 27 | 0.0000 |
| ENSRNOG00000007284 | Slc2a1 | 85 | 85 | 0.0000 |
| ENSRNOG00000007509 | Slc1a6 | 62 | 62 | 0.0000 |
| ENSRNOG00000007839 | Slc16a7 | 76 | 76 | 0.0000 |
| ENSRNOG00000007982 | Slc9a1 | 54 | 54 | 0.0000 |
| ENSRNOG00000008012 | Abcb1 | 88 | 88 | 0.0000 |
| ENSRNOG00000008169 | Slc24a2 | 18 | 18 | 0.0000 |
| ENSRNOG00000008243 | Slc43a1 | 14 | 14 | 0.0000 |
| ENSRNOG00000008376 | Slc2a3 | 77 | 77 | 0.0000 |
| ENSRNOG00000008479 | Slc8a1 | 23 | 23 | 0.0000 |
| ENSRNOG00000008557 | Abcb8 | 68 | 68 | 0.0000 |
| ENSRNOG00000008851 | Slc35b4 | 19 | 19 | 0.0000 |
| ENSRNOG00000008890 | Slc18a2 | 23 | 23 | 0.0000 |
| ENSRNOG00000009019 | Slc6a6 | 55 | 55 | 0.0000 |
| ENSRNOG00000009459 | Slc35f6 | 20 | 20 | 0.0000 |
| ENSRNOG00000009730 | CYP7B1 | 14 | 14 | 0.0000 |
| ENSRNOG00000009740 | Slco1c1 | 47 | 47 | 0.0000 |
| ENSRNOG00000009957 | Slc25a13 | 22 | 22 | 0.0000 |
| ENSRNOG00000010023 | Slc33a1 | 58 | 58 | 0.0000 |
| ENSRNOG00000010064 | Abcc4 | 77 | 77 | 0.0000 |
| ENSRNOG00000010210 | Slc7a11 | 62 | 62 | 0.0000 |
| ENSRNOG00000010592 | Slc25a27 | 15 | 15 | 0.0000 |
| ENSRNOG00000010597 | Slc5a7 | 63 | 63 | 0.0000 |
| ENSRNOG00000010830 | Slc25a4 | 48 | 48 | 0.0000 |
| ENSRNOG00000010938 | Slc7a10 | 50 | 50 | 0.0000 |
| ENSRNOG00000011184 | Slc13a4 | 84 | 84 | 0.0000 |
| ENSRNOG00000011361 | Slc37a4 | 36 | 36 | 0.0000 |
| ENSRNOG00000011455 | Slc36a4 | 27 | 27 | 0.0014 |
| ENSRNOG00000011677 | Slc39a10 | 34 | 34 | 0.0000 |
| ENSRNOG00000011929 | Abcd3 | 27 | 27 | 0.0000 |
| ENSRNOG00000012287 | Slc35e1 | 9 | 9 | 0.0000 |
| ENSRNOG00000012356 | Slc36a1 | 55 | 55 | 0.0000 |
| ENSRNOG00000012831 | Slc25a26 | 7 | 7 | 0.0000 |
| ENSRNOG00000012876 | Slc6a13 | 41 | 41 | 0.0000 |
| ENSRNOG00000012939 | Abca7 | 44 | 44 | 0.0000 |
| ENSRNOG00000013407 | Slc5a11 | 25 | 25 | 0.0000 |
| ENSRNOG00000013802 | Slc25a36 | 18 | 18 | 0.0000 |
| ENSRNOG00000014268 | Abca2 | 43 | 43 | 0.0000 |
| ENSRNOG00000014311 | Slc7a8 | 61 | 61 | 0.0000 |
| ENSRNOG00000014338 | Slc25a25 | 16 | 16 | 0.0000 |
| ENSRNOG00000014347 | Slc4a2 | 60 | 60 | 0.0000 |
| ENSRNOG00000014369 | Slc27a4 | 37 | 37 | 0.0000 |
| ENSRNOG00000014475 | Slc31a1 | 43 | 43 | 0.0000 |
| ENSRNOG00000014816 | Slc1a1 | 70 | 70 | 0.0000 |
| ENSRNOG00000014870 | Slc13a5 | 57 | 57 | 0.0000 |
| ENSRNOG00000015393 | Slc32a1 | 54 | 54 | 0.0000 |
| ENSRNOG00000015421 | Slc27a3 | 8 | 8 | 0.0000 |
| ENSRNOG00000015538 | Abcd2 | 26 | 26 | 0.0000 |
| ENSRNOG00000015741 | Slc2a13 | 70 | 70 | 0.0000 |
| ENSRNOG00000015948 | Slc1a5 | 80 | 80 | 0.0000 |
| ENSRNOG00000015971 | Slc12a2 | 75 | 75 | 0.0000 |
| ENSRNOG00000016147 | Slc17a6 | 32 | 32 | 0.0000 |
| ENSRNOG00000016163 | Slc1a3 | 71 | 71 | 0.0000 |
| ENSRNOG00000016414 | Slc22a17 | 14 | 14 | 0.0000 |
| ENSRNOG00000016753 | Slc14a1 | 15 | 15 | 0.0000 |
| ENSRNOG00000016827 | Slc38a3 | 74 | 74 | 0.0000 |
| ENSRNOG00000017188 | CYP27A1 | 24 | 24 | 0.0000 |
| ENSRNOG00000017210 | Slc22a23 | 10 | 10 | 0.0000 |
| ENSRNOG00000017302 | Slc6a3 | 34 | 34 | 0.0000 |
| ENSRNOG00000017693 | Slc2a5 | 69 | 69 | 0.0000 |
| ENSRNOG00000017993 | Abcb10 | 76 | 76 | 0.0000 |
| ENSRNOG00000018086 | Slc22a8 | 68 | 68 | 0.0013 |
| ENSRNOG00000018111 | Slc12a5 | 53 | 53 | 0.0000 |
| ENSRNOG00000018126 | Abca1 | 58 | 58 | 0.0000 |
| ENSRNOG00000018170 | Slc27a1 | 28 | 28 | 0.0000 |
| ENSRNOG00000018345 | Abce1 | 29 | 29 | 0.0000 |
| ENSRNOG00000018487 | Slc3a2 | 60 | 60 | 0.0000 |
| ENSRNOG00000018567 | Slc20a1 | 82 | 82 | 0.0000 |
| ENSRNOG00000018644 | Slc6a7 | 28 | 28 | 0.0000 |
| ENSRNOG00000018697 | Abcb6 | 36 | 36 | 0.0000 |
| ENSRNOG00000018746 | Slc30a5 | 31 | 31 | 0.0000 |
| ENSRNOG00000018785 | Slc16a13 | 57 | 57 | 0.0000 |
| ENSRNOG00000018824 | Slc7a5 | 68 | 68 | 0.0000 |
| ENSRNOG00000018863 | Abcc10 | 51 | 51 | 0.0000 |
| ENSRNOG00000018920 | Slc25a17 | 30 | 30 | 0.0000 |
| ENSRNOG00000019118 | Slc13a3 | 85 | 85 | 0.0000 |
| ENSRNOG00000019484 | Slc6a9 | 48 | 48 | 0.0000 |
| ENSRNOG00000019490 | Slc20a2 | 71 | 71 | 0.0000 |
| ENSRNOG00000019651 | Slc12a4 | 65 | 65 | 0.0000 |
| ENSRNOG00000019752 | Slc29a1 | 39 | 39 | 0.0000 |
| ENSRNOG00000019996 | Slc16a1 | 42 | 42 | 0.0000 |
| ENSRNOG00000020025 | Slc29a2 | 44 | 44 | 0.0000 |
| ENSRNOG00000020138 | Slc4a3 | 24 | 24 | 0.0000 |
| ENSRNOG00000020288 | Slc25a20 | 15 | 15 | 0.0000 |
| ENSRNOG00000020345 | Slc25a42 | 11 | 11 | 0.0000 |
| ENSRNOG00000020450 | Slc26a6 | 43 | 43 | 0.0000 |
| ENSRNOG00000020470 | Slc25a24 | 37 | 37 | 0.0000 |
| ENSRNOG00000020650 | Slc17a7 | 24 | 24 | 0.0000 |
| ENSRNOG00000020743 | CYP2S1 | 12 | 12 | 0.0000 |
| ENSRNOG00000020951 | Slc4a1 | 29 | 29 | 0.0000 |
| ENSRNOG00000021130 | Abcc8 | 56 | 56 | 0.0000 |
| ENSRNOG00000021262 | Slc23a2 | 47 | 47 | 0.0000 |
| ENSRNOG00000022305 | Abcc1 | 49 | 49 | 0.0000 |
| ENSRNOG00000022837 | Slc25a40 | 13 | 13 | 0.0000 |
| ENSRNOG00000022922 | Slc25a12 | 31 | 31 | 0.0000 |
| ENSRNOG00000024801 | Slc35a2 | 27 | 27 | 0.0000 |
| ENSRNOG00000025269 | Slc25a44 | 6 | 6 | 0.0000 |
| ENSRNOG00000025639 | Slc39a12 | 36 | 36 | 0.0000 |
| ENSRNOG00000026091 | Slc10a4 | 4 | 4 | 0.0000 |
| ENSRNOG00000027468 | Slc6a15 | 53 | 53 | 0.0000 |
| ENSRNOG00000027622 | Slc35e3 | 9 | 9 | 0.0003 |
| ENSRNOG00000028097 | Slc7a14 | 47 | 47 | 0.0000 |
| ENSRNOG00000028703 | Slc39a6 | 14 | 14 | 0.0000 |
| ENSRNOG00000028879 | Slc4a8 | 25 | 25 | 0.0000 |
| ENSRNOG00000029128 | CYP2D5 | 19 | 19 | 0.0000 |
| ENSRNOG00000029178 | Abcc5 | 91 | 91 | 0.0000 |
| ENSRNOG00000029871 | Slc8a3 | 14 | 14 | 0.0000 |
| ENSRNOG00000031662 | Slc6a5 | 36 | 36 | 0.0000 |
| ENSRNOG00000031824 | Slc44a2 | 5 | 5 | 0.0000 |
| ENSRNOG00000032261 | CYP2D6 | 26 | 26 | 0.0000 |
| ENSRNOG00000033164 | CYP2B3 | 25 | 25 | 0.0000 |
| ENSRNOG00000036678 | Aspscr1 | 6 | 6 | 0.0000 |
| ENSRNOG00000036693 | Slc25a10 | 33 | 33 | 0.0000 |
| ENSRNOG00000038001 | Slc25a1 | 37 | 37 | 0.0000 |
| ENSRNOG00000038398 | Slc25a31 | 27 | 27 | 0.0001 |
| ENSRNOG00000039980 | Slc25a5 | 38 | 38 | 0.0000 |
| ENSRNOG00000042320 | Slc41a1 | 3 | 3 | 0.0000 |
| ENSRNOG00000042496 | CYP4F5 | 19 | 19 | 0.0000 |
| ENSRNOG00000048450 | CYP4F37 | 16 | 16 | 0.0000 |
| ENSRNOG00000042731 | Slc25a18 | 5 | 5 | 0.0000 |
| ENSRNOG00000043513 | CYP4X1 | 15 | 15 | 0.0000 |
| ENSRNOG00000045821 | Slc41a3 | 29 | 29 | 0.0000 |
| ENSRNOG00000047493 | Slco1a4 | 29 | 29 | 0.0000 |
| ENSRNOG00000047781 | Slc25a23 | 26 | 26 | 0.0000 |
| ENSRNOG00000048487 | Slc12a9 | 44 | 44 | 0.0000 |
| ENSRNOG00000049944 | Slc25a22 | 12 | 12 | 0.0000 |
| ENSRNOG00000050057 | Abca3 | 43 | 43 | 0.0000 |
| ENSRNOG00000050090 | Slc6a17 | 46 | 46 | 0.0000 |
| ENSRNOG00000050860 | Abcb11 | 64 | 64 | 0.0000 |
| ENSRNOG00000055078 | CYP4B1 | 23 | 23 | 0.0000 |
| ENSRNOG00000066878 | CYP4A3 | 30 | 30 | 0.0000 |
| ENSRNOG00000068001 | Abcb4 | 69 | 69 | 0.0000 |

Note: protein_id: protein name, Symbol: protein symbol, degree: the degree of a node, all degree: the degrees of all nodes, DIA_Qvalue: protein identification Q-value.

**Table S6** Recovery and matrix effects of propranolol hydrochloride in plasma and brain tissue

| Samples | *C*/ng/g | Recovery rate | | Matrix effect | |
| --- | --- | --- | --- | --- | --- |
|  |  | Averages, % | RSD, % | Averages, % | RSD, % |
| Blood | 2.00 | 97.16 | 9.76 | 100.00 | 2.01 |
|  | 30.00 | 100.00 | 9.54 | - | - |
|  | 450.00 | 100.04 | 13.43 | 97.29 | 5.11 |
| Brain | 2.00 | 98.99 | 2.23 | 99.45 | 3.06 |
|  | 30.00 | 95.16 | 2.82 | - | - |
|  | 450.00 | 93.46 | 2.23 | 98.96 | 1.30 |

Note: C: drug concentration; ng/g: drug concentration in nanograms per gram of tissue.

**Table S7** Precision and accuracy of propranolol hydrochloride in biological samples

| Samples | Theoretical concentrations  (ng/ml) | Intra-day (n=5) | | | | Inter-day (n=3) | | | | |
| --- | --- | --- | --- | --- | --- | --- | --- | --- | --- | --- |
|  |  | Measured concentration  (ng/ml) | Precision  (RSD, %) | | Accuracy  (RE, %) | Measured concentration  (ng/g) | Precision  (RSD, %) | | Accuracy  (RE, %) | |
| Blood (ng/ml) | 1.17 | 1.18±0.14 | 11.47 | | 0.68 | 1.12±0.15 | | 13.24 | | -4.67 |
|  | 3.50 | 3.46±0.23 | 6.67 | | -1.27 | 3.47±0.17 | | 4.84 | | -0.87 |
|  | 30.00 | 28.61±1.81 | | 6.35 | -4.64 | 24.62±1.08 | | 4.39 | | -17.92 |
|  | 450.00 | 433.15±22.16 | 5.12 | | -3.74 | 450.31±18.21 | | 4.04 | | 0.07 |
| Brain  (ng/g) | 1.17 | 1.36±0.11 | 8.35 | | 15.91 | 1.07±0.08 | | 7.83 | | -8.56 |
|  | 3.50 | 3.57±0.22 | 6.21 | | 1.93 | 3.65±0.11 | | 2.94 | | 4.27 |
|  | 30.00 | 29.81±3.88 | 13.02 | | -0.63 | 26.55±1.17 | | 4.42 | | -11.51 |
|  | 450.00 | 428.04±9.37 | 2.19 | | -4.88 | 441.98±6.66 | | 1.51 | | -1.78 |

**Table S8** Stability of propranolol hydrochloride in plasma and brain tissue

| Samples | *C*/ng/g | 8h room temperature | -20℃ repeated freezing and thawing 3 times | -20℃30d |
| --- | --- | --- | --- | --- |
|  |  | RSD, % | RSD, % | RSD, % |
| Blood | 2.00 | 6.87 | 11.13 | 1.73 |
|  | 450.00 | 7.54 | 2.14 | 7.30 |
| Brain | 2.00 | 3.00 | 11.03 | 6.52 |
|  | 450.00 | 12.47 | 6.96 | 10.43 |

Note: C: drug concentration; ng/g: drug concentration in nanograms per gram of tissue.

**Table S9** Recovery and matrix effects of verapamil hydrochloride in plasma and brain tissue

| Samples | *C*/ng/g | Recovery rate | | | Matrix effect | | |
| --- | --- | --- | --- | --- | --- | --- | --- |
|  |  | Averages, % | | RSD, % | Averages, % | | RSD, % |
| Blood | 2.00 | 100.11 | 8.92 | | 100.03 | 6.20 | |
|  | 40.00 | 100.03 | 5.71 | | - | - | |
|  | 225.00 | 100.07 | 3.75 | | 100.03 | 6.13 | |
| Brain | 0.20 | 100.06 | 6.64 | | 97.32 | 3.66 | |
|  | 2.00 | 95.93 | 3.78 | | - | - | |
|  | 30.00 | 100.05 | 2.77 | | 99.15 | 1.63 | |

Note: C: drug concentration; ng/g: drug concentration in nanograms per gram of tissue.

**Table S10** Precision and accuracy of verapamil hydrochloride in biological samples

| Samples | Theoretical concentrations  (ng/ml) | Intra-day (n=5) | | | | | Inter-day (n=3) | | | | |
| --- | --- | --- | --- | --- | --- | --- | --- | --- | --- | --- | --- |
|  |  | Measured concentration  (ng/ml) | Precision  (RSD, %) | | Accuracy  (RE, %) | | Measured concentration  (ng/g) | Precision  (RSD, %) | | Accuracy  (RE, %) | |
| Blood  (ng/ml) | 1.17 | 1.12±0.06 | | 5.32 | | -4.21 | 1.30±0.07 | | 5.74 | | 10.87 |
|  | 3.50 | 3.51±0.14 | | 3.93 | | 0.17 | 3.65±0.22 | | 6.07 | | 4.16 |
|  | 40.00 | 40.92±1.91 | | 4.67 | | 2.29 | 36.48±0.81 | | 2.22 | | 8.81 |
|  | 225.00 | 254.59±1.56 | | 0.61 | | 13.15 | 256.71±4.18 | | 1.63 | | 14.09 |
| Brain  (ng/g) | 0.07 | 0.08±0.00 | | 6.04 | | 8.01 | 0.07±0.00 | | 1.37 | | -0.61 |
|  | 0.21 | 0.25±0.02 | | 6.36 | | 17.96 | 0.22±0.00 | | 0.93 | | 4.19 |
|  | 2.10 | 2.19±0.11 | | 5.14 | | 4.11 | 2.16±0.05 | | 2.10 | | 2.78 |
|  | 30.00 | 31.46±2.37 | | 7.53 | | 4.88 | 27.60±0.82 | | 2.95 | | -8.00 |

**Table S11** Stability of verapamil hydrochloride in plasma and brain tissue

| Samples | *C*/ng/g | 8h room temperature | -20℃ repeated freezing and thawing 3 times | -20℃30d |
| --- | --- | --- | --- | --- |
|  |  | RSD, % | RSD, % | RSD, % |
| Blood | 2.00 | 6.57 | 2.13 | 14.21 |
|  | 225.00 | 5.42 | 2.34 | 1.01 |
| Brain | 0.20 | 3.62 | 2.15 | 14.39 |
|  | 30.00 | 1.04 | 12.98 | 5.42 |

Note: C: drug concentration; ng/g: drug concentration in nanograms per gram of tissue.

**Table S12** Recovery and matrix effects of zolmitriptan in plasma and brain tissue

| Samples | *C*/ng/g | Recovery rate | | | Matrix effect | | |
| --- | --- | --- | --- | --- | --- | --- | --- |
|  |  | Averages, % | | RSD, % | Averages, % | | RSD, % |
| Blood | 2.00 | 100.06 | 5.45 | | 99.62 | 5.89 | |
|  | 40.00 | 99.80 | 3.82 | |  |  | |
|  | 225.00 | 98.49 | 3.87 | | 99.21 | 2.64 | |
| Brain | 0.20 | 96.45 | 4.06 | | 98.56 | 2.94 | |
|  | 2.00 | 100.01 | 3.44 | |  |  | |
|  | 30.00 | 97.54 | 3.41 | | 100.02 | 0.53 | |

Note: C: drug concentration; ng/g: drug concentration in nanograms per gram of tissue.

**Table S13** Precision and accuracy of zolmitriptan in biological samples

| Samples | Theoretical concentrations  (ng/ml) | Intra-day (n=5) | | | | | Inter-day (n=3) | | | | |
| --- | --- | --- | --- | --- | --- | --- | --- | --- | --- | --- | --- |
|  |  | Measured concentration  (ng/ml) | Precision  (RSD, %) | | Accuracy  (RE, %) | | Measured concentration  (ng/g) | Precision  (RSD, %) | | Accuracy  (RE, %) | |
| Blood  (ng/ml) | 1.17 | 1.30±0.15 | | 11.38 | | 10.92 | 1.26±0.10 | | 7.83 | | 7.55 |
|  | 3.51 | 3.10±0.11 | | 3.64 | | 0.13 | 3.55±0.02 | | 0.07 | | 1.43 |
|  | 40.00 | 43.79±4.16 | | 9.50 | | 9.48 | 39.32±0.94 | | 2.40 | | -1.70 |
|  | 450.00 | 429.57±12.81 | | 2.98 | | -4.54 | 432.37±9.00 | | 2.08 | | -3.92 |
| Brain  (ng/g) | 0.07 | 0.07±0.00 | | 2.76 | | 3.31 | 0.07±0.00 | | 4.61 | | 5.12 |
|  | 0.21 | 0.20±0.01 | | 6.04 | | -4.33 | 0.21±0.01 | | 5.42 | | 1.86 |
|  | 2.10 | 2.45±0.12 | | 4.93 | | 16.57 | 2.25±0.23 | | 10.01 | | 7.08 |
|  | 30.00 | 33.55±2.94 | | 8.77 | | 11.82 | 30.32±0.44 | | 1.46 | | 1.07 |

**Table S14** Stability of zolmitriptan in plasma and brain tissue

| Samples | *C*/ng/g | 8h room temperature | -20℃ repeated freezing and thawing 3 times | -20℃ 30d |
| --- | --- | --- | --- | --- |
|  |  | RSD, % | RSD, % | RSD, % |
| Blood | 2.00 | 14.36 | 4.74 | 1.91 |
|  | 450.00 | 8.15 | 8.53 | 5.74 |
| Brain | 0.20 | 2.80 | 2.48 | 2.29 |
|  | 30.00 | 2.79 | 4.27 | 1.25 |

Note: C: drug concentration; ng/g: drug concentration in nanograms per gram of tissue.

**Table S15** Summary of mRNA and protein expression levels of key drug transporters and metabolizing enzymes determined by DIA proteomics, Western blot, and RT-qPCR.

| Target | DIA (Protein) | Western Blot (Protein) | RT-qPCR (mRNA) | Overall Consistency |
| --- | --- | --- | --- | --- |
| Abcb1 | increase | increase | increase | Consistent |
| Abcg2 | decrease | increase | increase | Partially consistent |
| Slco1a2 | increase | increase | decrease | Partially consistent |
| CYP2B6 | decrease | decrease | decrease | Consistent |
| CYP2D6 | decrease | decrease | decrease | Consistent |
| CYP3A4 | increase | decrease | decrease | Partially consistent |

**Fig. S1.** Representative chromatograms for the pharmacokinetic investigation of hydrochloride propranolol, hydrochloride verapamil, zolmitriptan

Note: A(Ⅱ): hydrochloride propranolol, Ⅰ: mesylate hydrochloride, B(Ⅱ): hydrochloride verapamil, Ⅰ: Ubenimex, C(Ⅱ): zolmitriptan, Ⅰ: sumatriptan succinate, a: blank plasma sample, b: plasma containing the reference substances, c: plasma sample of rats after administration, d: blank brain tissue sample, e: brain tissue containing the reference substances, f: brain tissue sample of rats after administration.
