# Supplementary material for: Functional impact of high-altitude hypoxia on central nervous system drug transport: integrated analysis of blood-brain barrier permeability, drug transporter/enzyme expression, and pharmacokinetics
Source: Fluids Barriers CNS. 2026 Jun 11;23:100. doi: 10.1186/s12987-026-00829-y (PMC13501568; doi:10.1186/s12987-026-00829-y)
Supplement: Supplementary file 2 [file 12987_2026_829_MOESM2_ESM.docx]

**Fig. 5.** The protein expression of drug transporters, drug-metabolizing enzymes and tight-junction in blood-brain barrier and hCMEC/D_3_ cell under high-altitude hypoxia

Abcb1 170 kDa

Repeat 1	Repeat 2	Repeat 3

AH3

AH2

CH1

AH1

LC2

LC3

CH2

CH3

LC1

130

70

55

Abcg2 75 kDa

Repeat 1	Repeat 2	Repeat 3

LC3

LC2

LC1

AH3

CH2

CH1

AH1

AH2

CH3

70

55

40

35

Slco1a2 74 kDa

Repeat 1	Repeat 2	Repeat 3

130

70

LC2

AH1

CH3

AH3

LC3

CH2

AH2

CH1

LC1

55

Tjp1 225 kDa

Repeat 1	Repeat 2	Repeat 3

130

70

55

CH3

LC3

LC2

LC1

AH3

CH2

CH1

AH1

AH2

CYP2D1 52 kDa

Repeat 1	Repeat 2	Repeat 3

130

70

LC3

LC2

LC1

AH3

CH2

CH1

AH1

AH2

CH3

55

40

CYP2B1 55 kDa

Repeat 1	Repeat 2	Repeat 3

70

55

40

35

LC3

LC2

LC1

AH3

CH2

CH1

AH1

AH2

CH3

CYP3A1 52 kDa

Repeat 1	Repeat 2	Repeat 3

35

40

55

70

AH1

AH2

CH3

LC3

LC2

LC1

AH3

CH2

CH1

Actin 42 kDa

Repeat 1	Repeat 2	Repeat 3

LC3

LC2

LC1

AH3

CH2

CH1

AH1

AH2

CH3

35

130

40

70

55

ZO-1 225 kDa (hCMEC/D_3_ cell)

Repeat 1	Repeat 2	Repeat 3

LC3

LC2

LC1

AH3

CH2

CH1

AH1

CH3

AH2

70

120

Abcb1 170 kDa (hCMEC/D_3_ cell)

Repeat 1

Sequence: Normoxia, 2% hypoxia 24 h group, 5% hypoxia 24 h group, 10% hypoxia 24 h group, 2% hypoxia 2 h group, 2% hypoxia 6 h group, 2% hypoxia 12 h group, 2% hypoxia 24 h group, 2% hypoxia 48 h group

120

70

Repeat 2

Repeat 3

Abcg2 75 kDa (hCMEC/D_3_ cell)

Repeat 1

Sequence: Normoxia, 2% hypoxia 24 h group, 5% hypoxia 24 h group, 10% hypoxia 24 h group, 2% hypoxia 2 h group, 2% hypoxia 6 h group, 2% hypoxia 12 h group, 2% hypoxia 24 h group, 2% hypoxia 48 h group

120

70

Repeat 2

Repeat 3

Slco1a2 74 kDa (hCMEC/D_3_ cell)

Repeat 1

Sequence: Normoxia, 2% hypoxia 24 h group, 5% hypoxia 24 h group, 10% hypoxia 24 h group, 2% hypoxia 2 h group, 2% hypoxia 6 h group, 2% hypoxia 12 h group, 2% hypoxia 24 h group, 2% hypoxia 48 h group

120

70

Repeat 2

Repeat 3

CYP2D6 52 kDa (hCMEC/D_3_ cell)

Repeat 1

Sequence: Normoxia, 2% hypoxia 24 h group, 5% hypoxia 24 h group, 10% hypoxia 24 h group, 2% hypoxia 2 h group, 2% hypoxia 6 h group, 2% hypoxia 12 h group, 2% hypoxia 24 h group, 2% hypoxia 48 h group

55

70

Repeat 2

Repeat 3

CYP2B6 55 kDa (hCMEC/D_3_ cell)

Repeat 1

Sequence: Normoxia, 2% hypoxia 24 h group, 5% hypoxia 24 h group, 10% hypoxia 24 h group, 2% hypoxia 2 h group, 2% hypoxia 6 h group, 2% hypoxia 12 h group, 2% hypoxia 24 h group, 2% hypoxia 48 h group

70

55

Repeat 2

Repeat 3

CYP3A1 52 kDa (hCMEC/D_3_ cell)

Repeat 1

Sequence: Normoxia, 2% hypoxia 24 h group, 5% hypoxia 24 h group, 10% hypoxia 24 h group, 2% hypoxia 2 h group, 2% hypoxia 6 h group, 2% hypoxia 12 h group, 2% hypoxia 24 h group, 2% hypoxia 48 h group

55

Repeat 2

Repeat 3

Actin 42 kDa (hCMEC/D_3_ cell)

Repeat 1

Sequence: Normoxia, 2% hypoxia 24 h group, 5% hypoxia 24 h group, 10% hypoxia 24 h group, 2% hypoxia 2 h group, 2% hypoxia 6 h group, 2% hypoxia 12 h group, 2% hypoxia 24 h group, 2% hypoxia 48 h group

35

70

45

Repeat 2

Repeat 3
